# Construction of developmental lineage relationships in the mouse mammary gland by single-cell RNA profiling

**DOI:** 10.1038/s41467-017-01560-x

**Published:** 2017-11-20

**Authors:** Bhupinder Pal, Yunshun Chen, François Vaillant, Paul Jamieson, Lavinia Gordon, Anne C. Rios, Stephen Wilcox, Naiyang Fu, Kevin He Liu, Felicity C. Jackling, Melissa J. Davis, Geoffrey J. Lindeman, Gordon K. Smyth, Jane E. Visvader

**Affiliations:** 1 ACRF Stem Cells and Cancer Division, The Walter and Eliza Hall Institute of Medical Research, Parkville, VIC 3052 Australia; 2 Department of Medical Biology, The University of Melbourne, Parkville, VIC 3010 Australia; 3 Bioinformatics Division, The Walter and Eliza Hall Institute of Medical Research, Parkville, VIC 3052 Australia; 4 Australian Genome Research Facility, The Walter and Eliza Hall Institute of Medical Research, Parkville, VIC 3052 Australia; 5 Systems Biology & Personalised Medicine Division, The Walter and Eliza Hall Institute of Medical Research, Parkville, VIC 3052 Australia; 6 Department of Biochemistry and Molecular Biology, The University of Melbourne, Parkville, VIC 3010 Australia; 7 Department of Medicine, The University of Melbourne, Parkville, VIC 3010 Australia; 8 Parkville Familial Cancer Centre and Department of Medical Oncology, The Royal Melbourne Hospital and Peter MacCallum Cancer Centre, Parkville, VIC 3050 Australia; 9 School of Mathematics and Statistics, The University of Melbourne, Parkville, VIC 3010 Australia

## Abstract

The mammary epithelium comprises two primary cellular lineages, but the degree of heterogeneity within these compartments and their lineage relationships during development remain an open question. Here we report single-cell RNA profiling of mouse mammary epithelial cells spanning four developmental stages in the post-natal gland. Notably, the epithelium undergoes a large-scale shift in gene expression from a relatively homogeneous basal-like program in pre-puberty to distinct lineage-restricted programs in puberty. Interrogation of single-cell transcriptomes reveals different levels of diversity within the luminal and basal compartments, and identifies an early progenitor subset marked by CD55. Moreover, we uncover a luminal transit population and a rare mixed-lineage cluster amongst basal cells in the adult mammary gland. Together these findings point to a developmental hierarchy in which a basal-like gene expression program prevails in the early post-natal gland prior to the specification of distinct lineage signatures, and the presence of cellular intermediates that may serve as transit or lineage-primed cells.

## Abstract (web summary)

The mammary epithelium comprises two cell lineages but the heterogeneity amongst these during development is unclear. Here, the authors report single-cell RNA sequencing of the mouse mammary epithelium at four developmental stages, revealing diversity in both compartments and a transcriptional shift with puberty onset.

## Introduction

The mammary gland is a remarkably dynamic organ whose epithelium undergoes dramatic changes during morphogenesis and the reproductive cycle. Architecturally, the epithelium comprises two primary cellular lineages: an inner layer of luminal cells that surround the lumen and an outer layer of myoepithelial cells that lie in a basal position adjacent to the basement membrane. Cumulative evidence based on transplantation, colony-forming assays, and lineage tracing studies in mouse models indicates the presence of stem and committed progenitor cells that lie upstream of the mature epithelial cell types (myoepithelial, ductal luminal, and alveolar luminal) resident in the ductal tree^1, 2^. However, little is known about the spatio-temporal regulation of molecular pathways important for lineage specification in the mammary gland, thus highlighting the need for more refined transcriptional mapping studies.

Morphogenesis of the mammary gland occurs through distinct stages, with the majority of development taking place in the post-natal animal^3^. At birth, a rudimentary ductal tree exists and extends by allometric growth until puberty. During this stage, the epithelium undergoes massive expansion to form a highly elaborate and branched ductal tree that characterizes the adult gland. Ductal elongation and branching during puberty is largely driven by terminal endbuds (TEBs) located at the termini of the growing ducts. The gene expression portraits of different mammary epithelial cell types have been described at a population level^4–8^ but not at the single-cell level. Hence, a comprehensive understanding of heterogeneity within the different epithelial populations is lacking.

The global analysis of transcriptomes at the single-cell level has emerged as a powerful tool to understand cellular heterogeneity and genomic states. Such studies have provided valuable insights into lineage relationships, rare cellular subsets, and novel biomarkers for diverse organs. For example, single-cell RNA-seq (scRNA-seq) analysis of cerebral cortex cells from the developing brain^9^, developing heart^10^, the adult mouse forebrain^11^, lung epithelium^12^, intestinal cells^13^, olfactory neurons^14^, and pancreatic cells^15^ has revealed novel cellular subsets based on transcriptional and/or signaling pathways. Moreover, this methodology has been utilized to follow the induction of mouse embryonic fibroblasts to neuronal cells, identifying distinct intermediate stages during reprogramming^16^. The identification of lineage-primed or multipotent cells through single-cell analysis of haematopoietic^17, 18^, pancreatic^19^ and intestinal cells^20^ has provided important insights into rare cellular states.

Here we present comprehensive single-cell transcriptomes of epithelial cells in the post-natal mouse mammary gland at different developmental stages spanning pre-puberty, puberty, adulthood and pregnancy, as well as at different points of the estrus cycle. Transcript profiling was performed on two different platforms: the 10X Genomics Chromium System^21^ for large-scale analyses and the Fluidigm C1 platform for high-resolution sequencing. Determination and compilation of the transcriptomes of individual cells across distinct developmental stages revealed that a major transcriptional switch occurs at the onset of puberty from a relatively homogeneous to heterogeneous landscape. In the adult mammary gland, the luminal compartment was more stratified than the basal population, but rare basal subsets could be delineated. Interestingly, mixed-lineage intermediates poised towards a luminal fate were identified in purified basal cells of the adult as well as in pubertal and pregnant mammary glands. Collectively, these single-cell datasets spanning different developmental stages provide a valuable resource to decipher regulatory decisions in the mammary epithelium executed at the single-cell level.

## Results

### Reprogramming of the epithelial transcriptional landscape at puberty

**Fig. 1:**
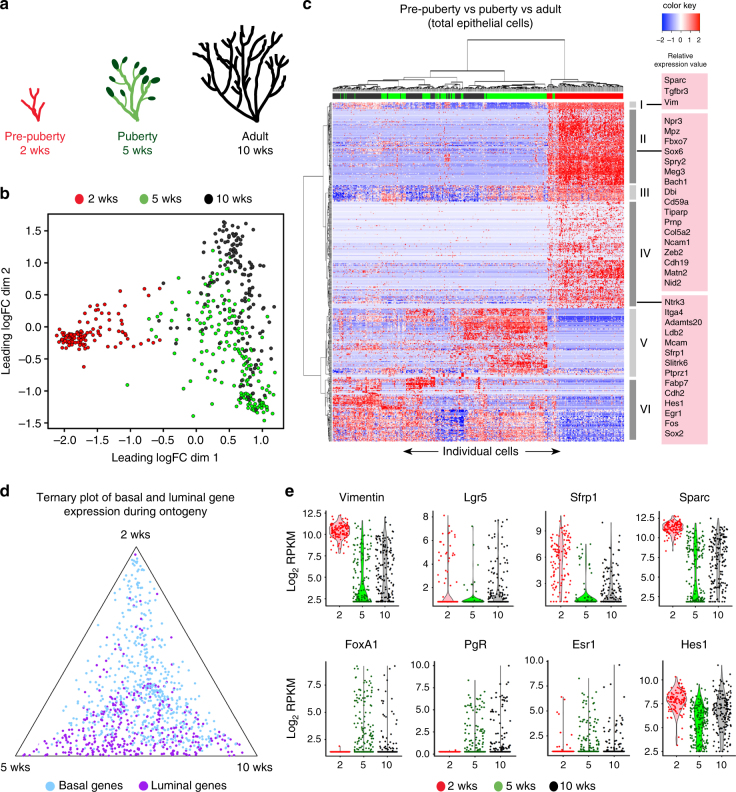
Acquisition of molecular heterogeneity in the mammary epithelium during puberty. **a** Schematic diagram of the mammary gland at pre-puberty (2 weeks; red), puberty (5 weeks; green, with TEBs in dark green) and adulthood (10 weeks; black). **b** Multidimensional scaling plot of scRNA-seq profiles. Total epithelial cells were collected at pre-puberty, puberty, and adulthood and profiled using the Fluidigm C1 platform. Distances on the plot correspond to leading log2-fold changes between cells. The first plot dimension (*x*-axis) clearly separates pre-pubertal cells from those in puberty or adult glands. Pre-puberty, 12 mice; puberty, 6 mice; adult, 4 mice per sort; three independent experiments. **c** Gene expression heatmap of the same 460 single cells shown in **b**. Rows correspond to the top 500 DE genes across the three developmental stages. Columns correspond to cells colored by stage as in **a** and **b**, indicated by bars at the top. Major gene clusters I to VI are highlighted in the side column; Supplementary Table 2 lists genes in these clusters. Selected genes are shown for clusters I, II, and IV, comprising genes specifically expressed at 2 weeks. Heatmap values are log expression scaled by row (red = high expression; blue = low expression). **d** Ternary plot showing relative expression of selected genes in the three developmental stages. For each gene, expression was averaged over all cells at each stage. Most genes known to be luminal-specific (purple) are strongly upregulated in cells from 5 and 10-week-old glands while different basal-specific genes (sky blue) are expressed at different developmental stages. Genes correspond to the top 1000 DE genes from Fu et al. 2015^22^. **e** Violin plots showing gene expression of selected genes for each cell by developmental stage. Vertical axis shows expression as log2-RPKM

To explore changes in cell diversity in the mammary epithelium during post-natal gland development (Fig. 1a), we performed scRNA-seq. We employed the Fluidigm C1 platform for single-cell capture to achieve in-depth expression profiling of individual mammary epithelial cells through different developmental stages (Supplementary Table 1). Combined with Illumina sequencing, this platform allowed us to assign up to 2 million read-pairs and to detect up to 8000 genes per cell (Supplementary Fig. 1a). The presence of a single cell per capture site was confirmed by direct visualization prior to sequencing.

We began by examining molecular heterogeneity in the mammary epithelium as the gland transitions from a rudimentary state in the early post-natal gland (2 weeks i.e. 14 days) to a developing tree in mid-puberty (5 weeks) and a highly branched ductal network in the adult (10 weeks) (Fig. 1a). An unsupervised plot of distances between the single-cell transcriptomes of 460 cells spanning the three developmental stages revealed a clear separation between pre-pubertal epithelial cells and those from pubertal or adult mice (Fig. 1b). A leading fold-change of more than 4-fold separates most pre-pubertal cells from the 5 or 10 week clusters. Pre-pubertal cells are relatively homogeneous whereas pubertal and adult cells are spread over a wider range and show more overlap (Fig. 1b). To understand the major genes driving these dramatic transcriptional differences, we clustered the cells using the most differentially expressed (DE) genes across the three developmental stages (Fig. 1c). Genes that were highly expressed in the pre-pubertal gland included the known basal genes *Vimentin (Vim), Ncam1*, the secreted matrix protein *Sparc*, the Wnt pathway modulator *Sfrp1*, and the transcriptional regulators, *Zeb2*, *Sox6*, *Egr1*, *Fos*, *Ldb2*, and *Bach1* (Gene clusters I, II, and IV in Fig. 1c; Supplementary Table 2). Gene cluster III included *Nras*, *Pten*, *Nf1*, and *Gsk3b* and is interesting in that it is expressed in both pre-pubertal cells and most pubertal cells but not in adult cells. Genes restricted to pubertal and adult mammary glands are discussed below.

To further interpret the lineage switches that occur upon ductal morphogenesis, we examined the behavior of genes identified in previous studies^22^ (Supplementary Table 3) to distinguish the basal/myoepithelial (denoted basal from hereon) and luminal lineages in the mammary gland. This revealed that basal gene expression occurs throughout all three developmental stages whereas expression of almost all luminal genes is confined to puberty and adulthood (Fig. 1d). Indeed, gene cluster V in the heatmap highlights the expression of definitive luminal genes (e.g., *Epcam, Keratin (Krt)8/18/19*) from puberty onwards (Fig. 1c; Supplementary Table 2). Violin plots of gene expression (Fig. 1e) illustrate that most pre-pubertal cells express abundant *Vim*, *Lgr5*, *Sfrp1*, *and Sparc* transcripts, whereas luminal lineage determinant genes such as the estrogen and progesterone receptors (*Esr1*, *PgR*) and *FoxA1* were expressed at low levels. By contrast, the Notch pathway effector gene *Hes1* was found in the majority of cells in 2-week-old mammary glands. Thus, a basal gene expression program prevails in pre-pubertal epithelial cells.

Interestingly, different sets of basal genes were expressed through pre-puberty, puberty, and adulthood, and a substantial subset was specific to early post-natal glands (Fig. 1c). To explore this further, we clustered individual cells according to their expression of basal and luminal lineage genes^22^ (Supplementary Fig. 1b). This confirmed that early post-natal cells were highly enriched for basal-specific genes, yet harbored a signature distinct from that of basal cells isolated from pubertal or adult glands (Supplementary Fig. 1b, c). Many cells in pre-puberty expressed elevated levels of basal-specific genes (e.g., *Sparc*, *Ngfr*, and *Erbb3*), whereas other basal genes were more highly expressed in pubertal and adult glands, such as the transcription factors *Trp63*, *Id4*, and *Snai2*, the Wnt inhibitory factor *Dkk3*, and myoepithelial contractile/cytoskeletal proteins including *Myh11* and *Acta2* (smooth muscle actin). These data are consistent with the presence of a unique basal-like gene expression program in the early post-natal gland (14 day old) prior to the establishment of distinct luminal and basal lineage-specific differentiation programs near the onset of puberty.

**Fig. 2:**
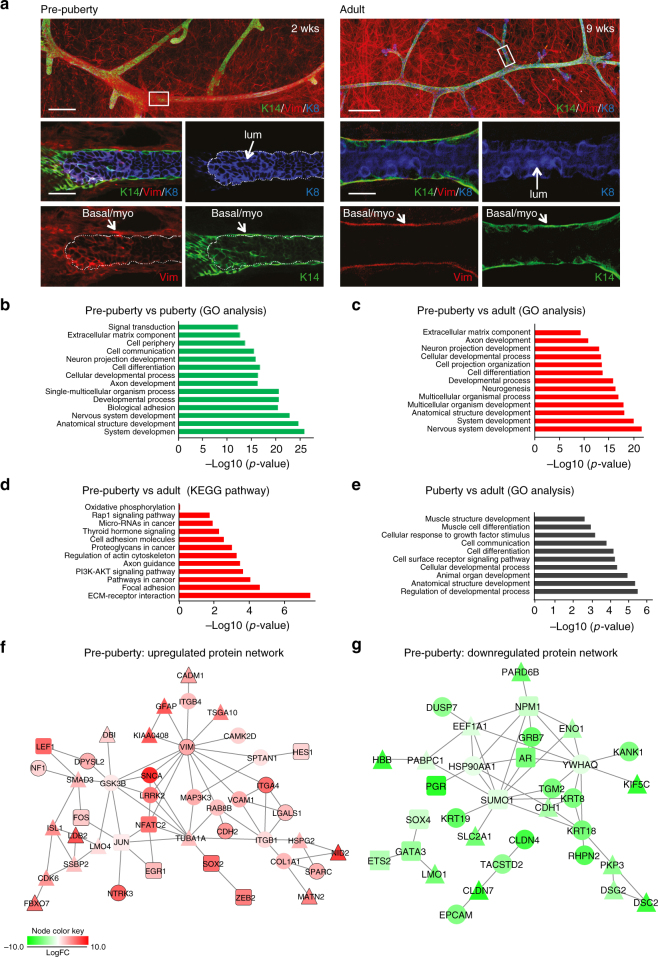
The majority of pre-pubertal epithelial cells exhibit a unique basal-like signature. **a** Whole-mount confocal 3D images (top panels) and optical sections (middle and bottom panels) of intact mammary ductal trees immunolabeled for Keratin 14 (green), Vimentin (red), and Keratin 8/18 (blue). Mammary glands were collected at 2 (left panels) and 9 weeks of age (right panels). Scale bars, 200 µm (top panels); 20 µm (bottom panels) (*n* = 3 mice per time point). Basal/myo indicates basal/myoepithelial cells in the outer layer. **b** Gene ontology terms upregulated in 2 vs. 5 week epithelial cells, based on differential expression analysis of the scRNA-seq profiles. **c** Gene ontology terms upregulated in 2 vs. 10 week epithelial cells. **d** KEGG pathways upregulated in 2 vs. 10 week epithelial cells. **e** Gene ontology terms downregulated at 5 vs. 10 weeks. **f** Protein interaction subnetwork of selected genes upregulated (red) in pre-pubertal cells relative to puberty. Node shape represents protein function (circle = signal transduction; square = transcription factor; rounded square = dual function, and triangle = none of the above). **g** Protein interaction subnetwork of selected genes downregulated (green) in pre-pubertal cells relative to puberty

Although the ductal tree is bilayered at 2 weeks, with distinct inner cuboidal cells and outer elongated cells, the largely homogeneous gene expression pattern of epithelial cells at the pre-pubertal stage suggests that they lie in an immature state. Indeed, confocal whole-mount 3D image analysis of pre-pubertal glands for expression of the canonical basal (Krt14 and Vim) and luminal markers (Krt8/18) revealed sporadic expression of basal proteins within the luminal layer of the distal ductal branches (Fig. 2a). Vimentin levels were examined as this is one of the most highly expressed genes in 2-week-old glands. The expression of basal markers in luminal cells is compatible with the scRNA-seq data that shows widespread expression of these genes across individual cells in the pre-pubertal gland. As anticipated, in the adult gland, Krt14 and Vimentin were exclusively expressed in the outer myoepithelial layer (Fig. 2a). Although E18.5^23, 24^ and newborn mammary glands^25^ frequently co-express Krt8 and Krt14, the ductal tree is not well-defined at these stages and does not comprise elongated myoepithelial cells^26^, which are evident by 2 weeks of age. Western blot analysis of individual cells^27^ from pubertal and adult mammary glands using the Milo system confirmed strong co-expression of Krt8/18 and Krt14 in ~13% of pre-pubertal epithelial cells but not in adult basal cells (Supplementary Fig. 1d).

### Predicted changes in protein interaction networks during ontogeny

Gene ontology and KEGG analyses of gene expression signatures derived for the different developmental stages showed that pre-pubertal cells were enriched for signaling pathways involved in developmental processes, anatomical structure, axon development, and cell projection organization relative to pubertal and adult epithelial cells (Fig. 2b–d). Interestingly, axon guidance molecules have been implicated in maintaining cell proliferation and adhesion during normal mammary gland development^28, 29^. Conversely, comparative analysis of 5 vs. 10 weeks revealed significant downregulation of developmental and anatomical processes and muscle differentiation pathways in pubertal cells (Fig. 2e).

We next integrated DE genes between the different developmental stages with protein–protein interaction data for the corresponding human orthologues (Methods section) to identify potential biological interactions that regulate the transition of epithelial cells through ontogeny. Our analysis focused on 1796 mouse genes (with 1673 human orthologues) that were DE (FDR < 0.001) between 2 and 5 weeks. We identified direct interactions among the proteins and annotated the resulting network with functional associations drawn from the gene ontology to identify parts of the network involved in signal transduction and transcriptional regulation, using Cytoscape analysis. The networks of transcription factors (TFs) and signal transducers (STs) that are DE between pre-puberty and puberty reveal potential mechanisms involved in driving ontogeny and are consistent with our previous studies^5^ (Fig. 2f, g; Supplementary Fig. 2a, b). Notably, the basal marker Vimentin, which is upregulated in pre-pubertal epithelial cells vs. those in puberty, forms a major hub in both the main network and the network of STs and TFs (Fig. 2f, Supplementary Fig. 2a). Interestingly, Vimentin interacts directly with other basal proteins highly expressed in pre-pubertal epithelium such as VCAM1, ITGB4/A4, CAMK2d, MAP3K3, and GSK3B, and the TF NFATC2. Upregulated networks implicated in indirect interactions via the vimentin node included several extracellular components (Collagen type 1, SPARC, Matrilins (MATN2)), the tyrosine kinase receptor NTRK3, as well as the TFs SOX2, ZEB2, FOS, EGR1, and SMAD3 (Fig. 2f). SMAD3 is also predicted to have direct links with a plethora of other transcriptional regulators that include ISL1, LEF1, LDB2, and LMO4. Taken together, the network analysis has highlighted the upregulation of multiple interacting proteins important for synthesis of extracellular matrix components and/or interactions with the matrix, thus pointing to a key role for the microenvironment in ductal development within the early post-natal gland.

Relative to puberty, the pre-pubertal gland was marked by downregulation of a protein interaction network interconnected by the shuttling nucleoprotein NPM1 and SUMO1 (Fig. 2g). SUMO1, which catalyzes the post-translational modification of proteins, including the activity of many TFs^30^, has been shown to bind Keratins and alter filament dynamics during cellular development^31^. Intriguingly, in this downregulated network, SUMO1 interacts with the luminal-specific androgen receptor, the luminal structural proteins KRT8, KRT18, and KRT19, and indirectly with E-CAD, the expression of which are very low until the onset of puberty (Fig. 2g). NPM1 is predicted to interact directly with SUMO, GRB7, the glycolytic enzyme ENO1, and the polarity complex protein PARD6B. Interrogation of a larger cohort of DE TFs and STs revealed additional information on interactions between known lineage-specific molecular regulators including the upregulated molecules ID2, MYOG, LEF1, ARNT, NGFR, and RARB, and the downregulated molecules GATA3, PGR, EHF, FGFR2, Klf5, AREG, ELF3, and FOXA1 (Supplementary Fig. 2a).

Comparison of puberty to adulthood showed relatively few genes that were DE at the very strict threshold we applied for network construction, and resulted in a smaller network than that observed for pre-puberty vs. puberty. Notable upregulated networks included NRAS/GRB10/GRB14/INHBA and GSK3B/CHD3/PLP1 nodes, while TF networks including FOS, MAFF, and the steroid hormone receptor PPARa were downregulated (Supplementary Fig. 2b).

### Dissection of heterogeneity in puberty and adulthood

**Fig. 3:**
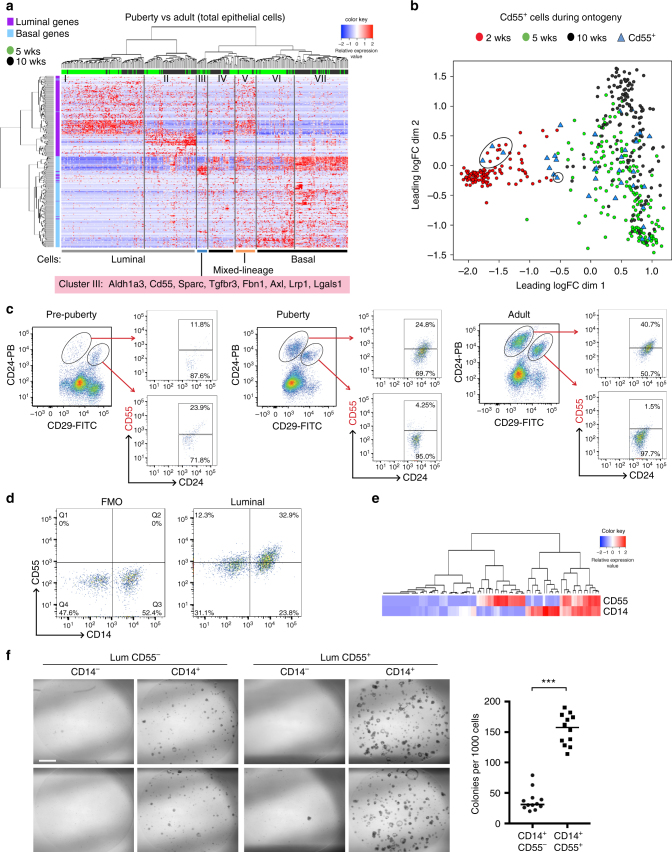
Identification of multiple subsets in pubertal and adult mammary glands. **a** Heatmap showing hierarchical clustering of 343 epithelial cells from pubertal (green) and adult (black) mammary glands based on their expression of lineage-specific genes (200 basal and 200 luminal genes; see Supplementary Table 3). Columns correspond to cells colored by stage, indicated by bars at the top. The cells shown are the same 5 and 10-week-old cells as in Fig. 1b, c and Supplementary Fig. 1b, c. Seven major cell clusters are indicated (gene lists shown in Supplementary Table 4). Basal genes = blue; luminal genes = purple; red = high expression; blue = low expression. **b** Multidimensional scaling plot of scRNA-seq profiles. These are the same as in Fig. 1b but cells expressing *Cd55* (RPKM > 40) are highlighted as blue triangles. *Cd55* ^*+*^ cells are present at all three stages but pre-pubertal glands harbor only rare cells (circled in the plot). **c** CD55 expression within epithelial subsets isolated from mammary glands in pre-puberty (2 wks) (left), puberty (5 weeks) (center), and adult (10 weeks) (right). Representative dot plots of CD29 vs. CD24 expression in Lin^–^ cells (see gating strategy in Supplementary Fig. 3) are shown on the left, while CD29 vs. CD55 expression in either the luminal (top) or basal (bottom) compartment is shown on the right (*n* = 3 independent experiments per time-point: 12 mice per experiment for pre-puberty, 6 mice per experiment for puberty, and 4 mice per experiment for adult). **d** The CD14/CD55 flow cytometric plots of freshly sorted Lin^–^CD29^lo^CD24^+^ (luminal) mammary epithelial cells isolated from 10-week-old FVB/N mice (representative of 5 independent experiments, 4 mice per experiment). **e** Hierarchical clustering of 76 purified luminal cells based on *Cd55* and *Cd14* gene expression (Fluidigm C1 platform; see Supplementary Fig. 4c). Red = high expression; blue = low expression. **f** Clonogenic activity of CD14^–^CD55^–^, CD14^–^CD55^+^, CD14^+^CD55^–^, and CD14^+^CD55^+^ luminal (CD29^lo^CD24^+^) subsets from 10-week-old FVB/N mice, plated in Matrigel (1000 cells per population). Representative of three independent experiments, 4 mice per experiment. *P*-value (unpaired *t*-test) <0.0001. Scale bar; 1000 µm

To interrogate heterogeneity in pubertal and adult glands in more detail, we excluded pre-pubertal cells and re-clustered epithelial cells from these two stages according to their expression of defined basal and luminal lineage genes^22^ (Supplementary Table 3). Hierarchical clustering revealed seven major cell clusters (Fig. 3a; Supplementary Table 4). As expected, both luminal cells (clusters I and II) and basal cells (clusters VI and VII) could be clearly identified at these stages, in contrast to the homogeneous basal-like cells evident in pre-pubertal glands (Supplementary Fig 1b). Clusters I and II were enriched for definitive markers of mature luminal (ML) and luminal progenitor cells, respectively. Conversely, cluster III was enriched for basal genes (*Axl*, *Sparc*, *Tgfbr3*, and *Lrp1*)*, Cd55* and the progenitor gene *Aldh1a3* (Fig. 3a), and was more prevalent in pubertal mammary glands. Cluster V was also mainly found amongst pubertal cells and expressed a subset of both basal and luminal genes, indicative of a mixed-lineage subpopulation. Cluster IV consisted mostly of adult cells and expressed a subset of basal genes. In a recent report, scRNA-seq of 5-week-old pubertal glands indicated that TEB-enriched cells largely cluster into basal and luminal groups despite their heterogeneous expression profile^32^, consistent with findings presented here. Interestingly, close examination of the basal cell clusters VI and VII revealed that cluster VI mainly comprised pubertal cells, whereas cluster VII was primarily found amongst adult basal cells. Moreover, a higher proportion of pubertal cells expressed a gene subset that included *Snai2, Moxd1, Nrg1, Wif1*, and *Bmp7* (Supplementary Table 4; bottom of cluster VI). It is plausible that these cells correspond to the cap cells of TEBs, which characterize the pubertal gland.

### CD55 marks distinct populations through development

To further explore features of cluster III (Fig. 3a), we selected Cd55 since this gene encodes a cell surface marker that enables flow cytometry to track protein expression during development. CD55 has been shown to be a discriminating marker of one of the earliest branch-points in the hematopoietic system^18^, so we returned to pre-pubertal cells to determine whether *Cd55*-expressing cells are already present at this stage. Intriguingly, a small number (4 out of 117) of cells from 2-week-old glands expressed *Cd55* transcripts (Fig. 3b). Flow cytometry (gating strategy shown in Supplementary Fig. 3) showed CD55 expression in a minority of cells at the pre-pubertal stage but in larger numbers of pubertal and adult mammary epithelial cells (Fig. 3c), compatible with the scRNA-seq data. In the pre-pubertal gland, CD55^+^ cells were primarily localized to the basal-like population, but during development, CD55 expression shifted towards the luminal lineage. Indeed, CD55^+^ cells were exclusively found in the luminal compartment of the adult gland and comprised approximately half of the Lin^*–*^CD29^lo^CD24^+^ population (Fig. 3d). CD55^+^ luminal cells from pubertal or adult tissue harbored 3.0- or 3.3-fold higher colony-forming capacity than CD55^–^ luminal cells, respectively, suggesting that CD55 enriches for progenitor cells.

Comparison of CD55 levels relative to that of another adult luminal progenitor marker CD14^4, 33^ revealed that adult mammary glands comprised ~33% double-positive cells in the luminal fraction (Fig. 3d), compatible with the overlap observed at the messenger RNA level (Fig. 3e). Colony-forming assays in Matrigel revealed that CD14^+^CD55^+^ cells were significantly enriched for clonogenic activity (Fig. 3f), thus reflecting a highly purified population of committed progenitor cells. The large acinar colonies are consistent with the presence of luminal progenitor cells. Collectively, these observations suggest that CD55 expression is first acquired by rare basal-like cells prior to the onset of puberty and that it subsequently demarcates a potent progenitor cell that becomes restricted to a luminal cell fate.

### Large-scale scRNA-seq identifies luminal intermediates in the adult

**Fig. 4:**
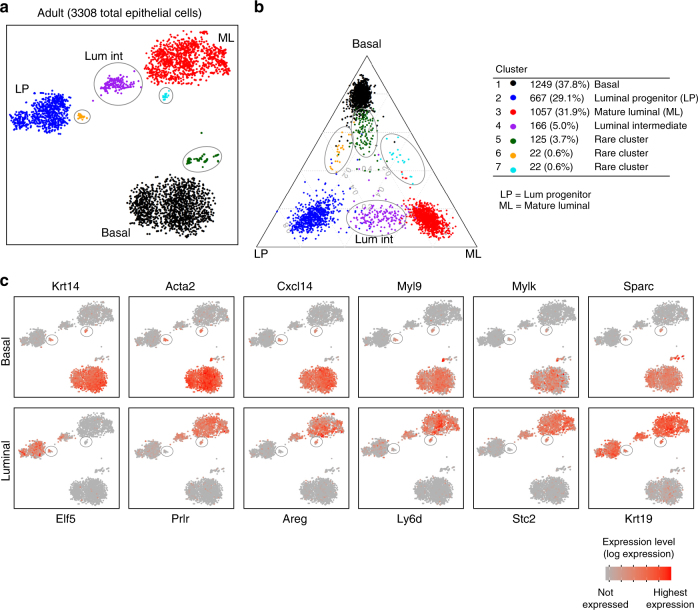
Transcriptome analysis of 3308 adult total epithelial cells reveals a luminal intermediate cluster. **a** t-SNE plot of the transcriptomes of total epithelial cells from adult mammary glands (*n* = 4 mice) generated using the 10X Genomics Chromium platform. The major clusters could be identified as basal (cluster#1, 1249 cells), luminal progenitor (LP; cluster #2, 667 cells), mature luminal (ML; cluster #3, 1057 cells), luminal intermediate (Lum Int; cluster #4, 166 cells), and cluster #5 (green, 125 cells), as indicated. **b** The ternary plot classifies total single epithelial cells into basal, LP, and ML cell clusters based on known lineage-specific signatures for these cell types. Cells are plotted according to the relative proportion of genes they express that are specific to the three cell types. This analysis also showed the presence of the Lum Int cluster (purple cells) between LP and ML cells. **c** The epithelial cell clusters were interrogated for the expression of typical basal and luminal genes to determine their patterning amongst the cell clusters. Color indicates expression level in log2-CPM

To investigate molecular heterogeneity more fully within the mammary epithelial compartment, we used the 10X Genomics Chromium platform to profile gene expression in thousands of individual cells (at lower sequencing depth than provided by the Fluidigm C1 technology) (Supplementary Table 1). 10X profiling of 3308 individual epithelial cells (Lin^–^CD24^+^) from adult mice revealed three major clusters as well as several satellite clusters (clusters 4–7) (Fig. 4a) based on t-distributed stochastic neighbor embedding (t-SNE)^34^. To uncover the lineage identity of each cluster, we overlaid the t-SNE clusters onto a ternary plot with each cell positioned according to its expression of known markers for mammary stem cell (MaSC)/basal (basal), luminal progenitor (LP), and ML cell types^8^ (Fig. 4b). The ternary plot shows the proportion of basal, LP, and ML-specific genes expressed by each cell and recapitulates the t-SNE clusters to a striking degree. Expression analysis of the cell clusters for basal genes (*Krt14*, *Acta2*, *Cxcl14*, *Sparc*, *Mylk*, and *Myl9)*, and luminal genes (*Krt19, Areg*, the prolactin receptor (*Prlr)*, the estrogen-regulated secreted glycoprotein *Stc2*, the antigen *Ly6d*, and the progenitor marker *Elf5)* confirmed the identity of the major clusters (Fig. 4c). These data indicated three luminal clusters and a largely homogeneous basal population associated with a small cluster (#5) that is closely aligned with the basal population at the molecular level (Fig. 4a, b). The genes most highly expressed in this cluster relative to basal cells are shown in Supplementary Fig. 4a. Other very rare clusters (#6 and 7) apparent in Fig. 4a may represent different regulatory states, although this remains to be determined. These clusters were noted to express basal as well as selective luminal genes including the epidermal growth factor (EGF) family member amphiregulin (*Areg), Prlr* and *Krt19* (discussed further below). Analysis of sorted luminal cells on the 10X platform confirmed the presence of three luminal populations and did not reveal mixed-lineage clusters (data not shown).

In the context of the luminal lineage, a novel cluster (#4) was identified to lie between the two major luminal populations in both the t-SNE and ternary plots (Fig. 4a, b). Interestingly, this was found to express the majority of genes at an intermediate level between that in progenitor (LP) and mature (ML) cells (Supplementary Fig. 4b). We therefore denoted this cluster Luminal Intermediate or Lum Int. Of 3711 genes that were DE between the LP, Lum Int, and ML cell clusters (FDR < 0.01), more than 88% showed steadily increasing or decreasing expression from LP to Lum Int to ML, further supporting the interpretation of the Lum Int population as an intermediate state between LP and ML (Supplementary Fig. 4b). In addition, a small number of genes (e.g., *Jund*, *Irx5*, *Sox4*, *Ncor1*, and *Igfbp2/6*) showed at least 20% higher average expression in the Lum Int population relative to either LP or ML. Collectively, these findings suggest that Lum Int cells represent a transit population between the progenitor and differentiated states, rather than a distinct type of luminal cell. As anticipated, intermediate luminal cells were detected amongst purified luminal cells (Lin^–^CD29^lo^CD24^+^) from adult mammary glands upon interrogation with the gene expression profiles of LP and ML cells^5, 8^ (Supplementary Fig. 4c). Finally, the Lum Int cluster was readily demarcated by scRNA-seq of total mammary epithelial cells (5387) isolated from pubertal glands (Supplementary Fig. 5a, b). Therefore, this intermediate population appears to arise in puberty, when definitive commitment to the luminal lineage is first observed.

### Deep scRNA-seq of basal cells uncovers mixed-lineage cells

**Fig. 5:**
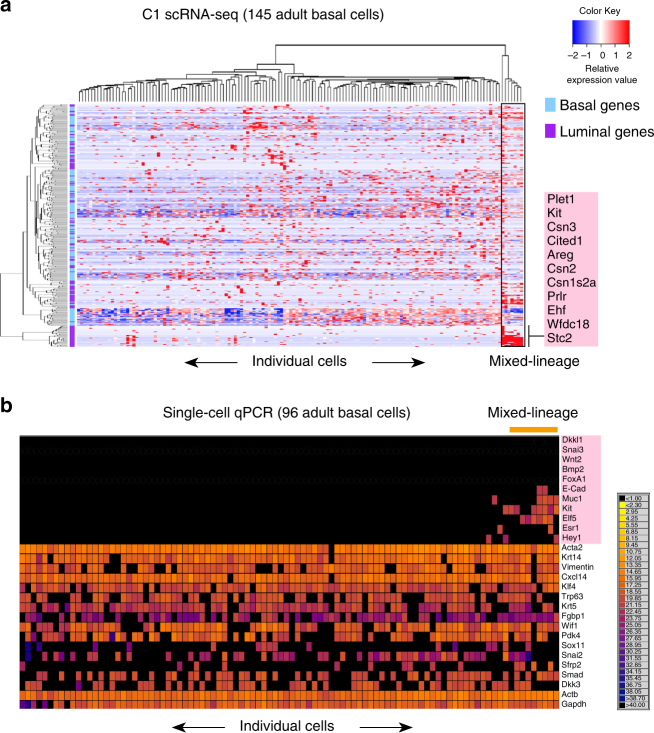
Purified basal epithelial cells in the adult gland comprise a lineage-primed subset. **a** Heatmap showing expression of the top 200 DE basal (blue) and top 200 DE luminal (purple) lineage genes in 145 sorted basal epithelial cells (Lin^–^CD29^hi^CD24^+^) from adult mammary glands that were captured on the Fluidigm C1 platform (red = high expression, blue = low expression) (*n* = 4 mice). Mixed-lineage intermediate cells are marked in the box. **b** Heatmap showing qRT-PCR expression for 96 sorted adult basal cells (*n* = 6 mice; 4 independent experiments). Taqman assays for 13 basal and 11 luminal lineage genes were applied using the Fluidigm Biomark multiplexed qPCR platform. The color key shows Ct values from black (Ct > 40, no expression) to yellow (highest expression). Mixed-lineage cells expressing both basal and luminal genes are marked by the orange bar

High-resolution transcriptome mapping (Fluidigm C1) of 145 purified basal cells (Lin^–^CD29^hi^CD24^+^) from the adult mammary gland confirmed a largely dominant basal population, as also observed by 10X Chromium scRNA-seq analysis of 4754 sorted basal cells (Supplementary Fig. 5c). Interestingly, clustering of the C1 platform-based single-cell transcriptomes of basal cells according to basal and luminal lineage genes indicated the presence of mixed-lineage cells (Fig. 5a). These cells comprised ~5% of the basal compartment, with 7 out of 145 cells expressing luminal genes such as *Kit, Areg, Prlr*, the TFs *Ehf* and *Cited1*, and the milk protein genes *Csn1s2a, Csn2*, and *Csn3*, in addition to core basal genes. Such cells, albeit rare, were also evident amongst total epithelial cells (Fig. 3a; Supplementary Fig. 1b). Additional evidence for the existence of a lineage-primed population derived from the analysis of 96 sorted basal cells using the Fluidigm Biomark Real-Time PCR system^35^. These basal cells were found to express the luminal genes *Elf5*, *Muc1*, *Kit*, *Esr1*, and/or *E-cad* (Fig. 5b), implying the presence of a transient subpopulation that may be primed for luminal lineage commitment. Conversely, interrogation of purified adult LP and mature cells for basal gene expression did not reveal basal lineage-priming in these cells by multiplex quantitative reverse transcription PCR (Supplementary Fig. 6a, b). Together, these data suggest that the basal state may precede commitment to a luminal cell fate in the post-natal mammary gland.

### Mixed-lineage cells exist at other developmental stages

A larger mixed-lineage population was evident in pubertal compared to adult mammary glands. This was first apparent in the developmental analysis across three stages, which revealed cells with dual-lineage features in puberty and adulthood but not in the pre-pubertal phase (Fig. 3a; Supplementary Fig. 1b, c). The variable expression patterns evident in this mixed-lineage population (Cluster V in Fig. 3a) indicate that individual cells express distinct combinations of basal and luminal genes, compatible with the properties of lineage-primed cells. Mixed-lineage clusters (#6 and 7) were also observed amongst pubertal mammary epithelial cells captured on the 10X Chromium platform (Supplementary Fig. 5a). These cells expressed core basal genes including *Krt14*, *Acta2*, *Myl9*, *Sparc*, *Mylk*, and/or *Cxcl14* and luminal genes such as *Areg*, *Prlr*, *Krt19*, *Cited1*, and/or *Stc2*.

To investigate whether mammary glands during pregnancy comprised an intermediate subset, total epithelial cells isolated at mid-pregnancy (12.5 days) were subjected to high-resolution scRNA-seq analysis. While the multidimensional scaling (MDS) plot (Supplementary Fig. 6c) showed dispersed cells, hierarchical clustering of cells according to lineage-specific genes revealed the presence of an expanded mixed-lineage intermediate that expressed canonical basal genes together with numerous luminal genes (*Elf5*, *Krt8*, *Cyp24a1*, *Wap*, *Csn1s2b*, *Csn2*, and *Lalba*) (Supplementary Fig. 6d). These findings suggest that basal cells are primed towards the alveolar lineage and milk protein production in pregnancy to enable expansion and differentiation in preparation for lactation. A small cluster highly enriched for ML cell-specific genes such as *Prlr*, *Cited1*, *Esrrb*, and *Cxcl15* was also noted.

### Global changes in gene expression during the estrus cycle

**Fig. 6:**
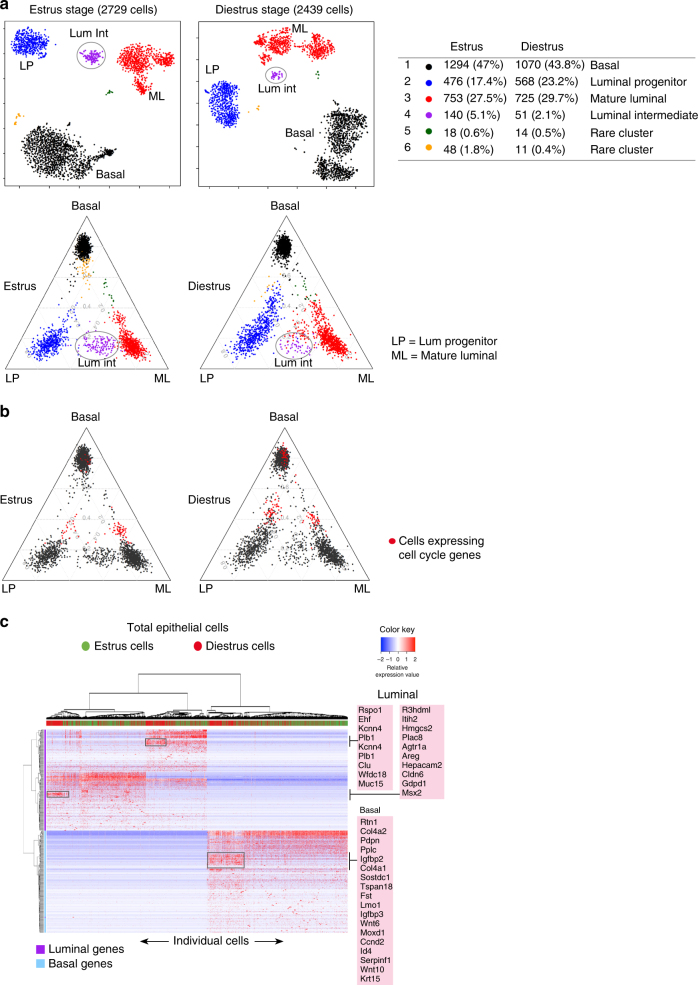
Transcriptome analysis of estrus and diestrus adult epithelial cells reveals specific changes in molecular heterogeneity. **a** t-SNE plots of the transcriptomes of total epithelial cells (Lin^–^CD24^+^; *n* = 2 mice for each phase) from adult mammary glands staged at estrus (2729 cells) and diestrus (2439 cells) were generated using the 10X Genomics Chromium platform (top panels). The major epithelial clusters (1–3; see table on right) representing basal, mature luminal (ML) and luminal progenitor (LP) cell populations could be identified by ternary plots based on known lineage-specific signatures for these cell types (bottom panels). Cells are plotted according to the relative proportion of genes they express that are specific to the three cell types. Cluster 4 depicts the Lum Int (luminal intermediate) population. **b** Ternary plots as for **a** but with cells marked by expression of cell cycle genes. Cells marked red belong to the cell clusters with cell cycle upregulation identified in Supplementary Fig. 7. **c** Heatmap showing hierarchical clustering of all estrus (green) and diestrus (red) epithelial cells together based on their expression of lineage-specific signature genes (200 DE basal and 200 DE luminal genes). The luminal and basal gene clusters specifically upregulated in diestrus stage are marked by boxes in the side columns (basal genes = blue; luminal genes = purple; red = high expression; blue = low expression)

The mouse mammary gland undergoes hormonal cycling every 3–5 days^36^. To evaluate gene expression changes that accompany cycling at the single-cell level, we collected mammary glands at different phases of the estrus cycle. Estrus and diestrus represent the luteal and follicular phases of the hormonal cycle, respectively, whereby the diestrus phase is characterized by maximal progesterone levels, and estrus by a surge of circulating estrogen^37^. Individual adult mice were staged by vaginal cytology at the time of mammary gland collection and glands from two mice subsequently pooled for estrus and diestrus. scRNA-seq of 2729 total epithelial cells in estrus and 2439 cells in diestrus using the 10X Chromium platform indicated the presence of the three major populations (basal, LP, and ML) (Fig. 6a). However, the Lum Int population (cluster 4) was substantially diminished in diestrus whereas the LP population was unaltered, suggesting a rapid transit to a more differentiated state. Moreover, the ML population appeared to segregate into two subsets in diestrus, and one of the clusters tightly associated with ML signature genes such as PgR^38, 39^. Given that progesterone is a potent mitogen and is elevated in diestrus, we examined the expression of cell cycle genes including Ki67. A notable increase in the number of cycling/proliferative cells was observed in the basal population in diestrus (Fig. 6b; Supplementary Fig. 7a, b), compatible with previous findings that the MaSC-enriched/basal population is expanded during diestrus or upon treatment with progesterone^40^.

Hierarchical clustering of the scRNA-seq profiles of combined estrus and diestrus cells with respect to the top DE basal and luminal genes further highlighted that changes across the stages were not widespread but more localized (Fig. 6c). Three specific cell clusters of upregulated genes could be identified in diestrus. The upregulated basal genes included *cyclin D2*, the ligands *Wnt6, Wnt10* and basal transcriptional regulator *Id4*, while luminal genes included *Areg* and Rspondin1 (*Rspo1*), all of which are compatible with the increase in proliferation that occurs in this phase (Fig. 6c).

## Discussion

**Fig. 7:**
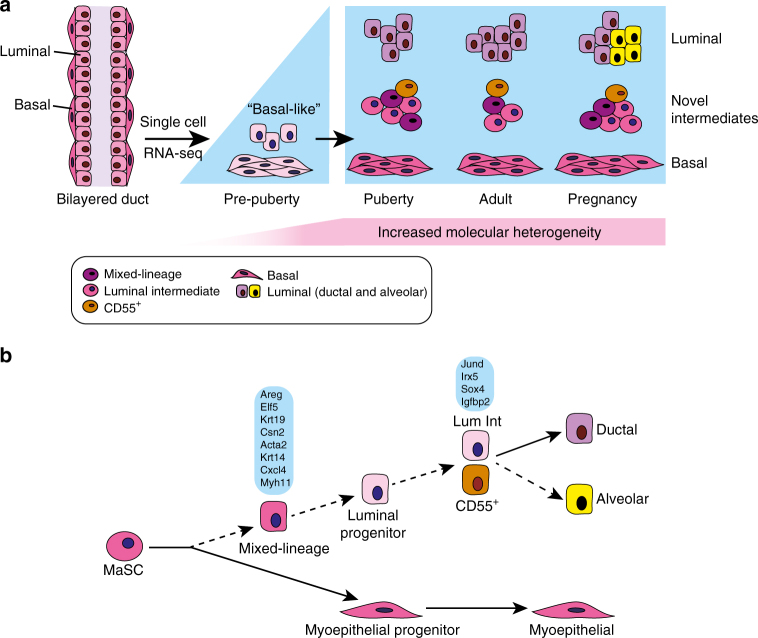
Schematic model depicting lineage relationships and an increase in molecular and cellular heterogeneity during mammary gland ontogeny. **a** A profound shift in gene expression occurs between pre-puberty and the onset of puberty. New intermediate clusters identified include a luminal intermediate (Lum Int) population and a mixed-lineage or ‘lineage-primed’ subset. Lum Int expresses luminal genes at levels intermediate between LP and ML, but a small number of genes (four indicated) showed at least 20% higher average expression. CD55^+^ cells with intermediate properties were readily detectable in puberty and become restricted to the luminal lineage during development. **b** Proposed position of the newly identified clusters along the epithelial hierarchy. The mixed-lineage subset within the basal compartment may represent a transient population that precedes commitment to the luminal lineage. Molecular evidence places the Lum Int cluster between the luminal progenitor and mature ductal/alveolar cells

Unmasking cellular heterogeneity within a given organ is critical for understanding the biology of normal and diseased tissues. Here we provide high-resolution single-cell transcriptomes of mammary epithelial cells at different stages of ontogeny as well as large-scale analyses of the adult mammary epithelium, which have enabled the construction of a temporal lineage specification map. The developmental phase in the post-natal animal was found to profoundly affect cellular and genetic heterogeneity (Fig. 7). Strikingly, epithelial cells in the early post-natal gland exist in a unique molecular state that is reminiscent of basal cells. These cells exhibit a relatively homogeneous transcriptional landscape, which is distinct from that of adult basal cells and show little luminal gene expression. Curiously, luminal cells that line the bilayered ducts in 2-week-old glands display the molecular characteristics of basal-like cells, suggestive of an immature state. Near the onset of puberty, a major transcriptional reprogramming event occurs, with the emergence of cells that harbor distinct lineage-specific gene signatures. Thus, the overall patterning suggests a branched trajectory of development with progression from a basal-like population in the early post-natal gland towards a more diverse array of lineage-specific epithelial cells from puberty into adulthood.

The basal compartment of the mammary gland appears to be dynamically regulated through the different stages of development in the post-natal animal. In the pre-pubertal gland, the basal-like gene expression signature differs substantially from that of basal cells in pubertal and adult mammary glands, although a few genes were common across the three stages. Moreover, the composition of the basal compartment was similar but not identical between pubertal and adult glands. High-resolution single-cell transcriptome analysis of purified basal cells from adult mice revealed an apparent continuum of cells that exhibited strong basal character. Nevertheless, hierarchical clustering uncovered a rare basal subset that displayed ‘mixed-lineage’ features, namely the expression of canonical basal genes together with numerous luminal genes such as *Elf5*, *Csn2*, *Areg*, *Cd14*, and *Prlr*.

The mixed-lineage or ‘lineage-primed’ cluster in the basal compartment was observed at different developmental stages including puberty, adulthood, and pregnancy. These cells, however, were absent in 2-week-old mammary glands and thus appear to coincide with the increased epithelial diversity that emerges at the onset of puberty. Although scarce amongst basal cells in the steady-state adult mammary gland, mixed-lineage cells were more prevalent in puberty and pregnancy, commensurate with the massive epithelial expansion that accompanies these stages. Their presence in mid-pregnancy is consistent with microarray analysis of the bulk basal cell population at day 12.5^41^. The detection of mixed-lineage or lineage-primed intermediates across different developmental stages suggests that transcriptional priming may not be stochastic. It is presumed that these cells represent a transient population that is poised for commitment to the luminal lineage, reminiscent of ‘lineage-primed’ stem and progenitor cells initially reported in the haematopoietic system^18, 42–44^. Indeed, only luminal and not myoepithelial/basal cells express milk protein genes at the protein level, consistent with the existence of transcriptionally-primed cells. Given that highly purified basal cells appear to comprise lineage-primed cells, it is presumed that these cells are descendants of basal epithelial cells, although the possibility remains that luminal cells could transition to a basal-like state under certain conditions. The demonstration that a single basal cell has the capacity to reconstitute an entire ductal tree upon transplantation into the fat pad^45, 46^ is consistent with the notion of ‘primed’ multipotent cells. Notably, single-cell RNA analyses have identified lineage-primed cells in the haematopoietic system^17, 18^, intestine^20^, and pancreas^19^, as well as heterogeneity in the haematopoietic stem cell pool^47^.

In the luminal compartment of the adult mammary gland, three distinct luminal clusters were visualized through interrogation of single-cell transcriptomes. These corresponded to a novel luminal intermediate cluster (Lum Int) as well as the expected LP and ML populations, which largely reflect differentiation status. Moreover, in contrast to the ML subset, LP cells are largely hormone receptor-negative. The novel cluster forms a prominent population that exhibits properties intermediate between that of the LP and ML subsets, and likely represents a transit population. Interestingly, this cluster was reduced in size in diestrus, perhaps indicating a faster transit time between the progenitor and mature states in response to higher serum progesterone levels. Indeed, the ML population, which expresses high levels of *Esr1* and *Pgr* ^7, 8, 48^, was more pronounced in diestrus than estrus.

CD55 was identified as a novel marker of early progenitor cells. Rare CD55^+^ cells could be discerned in the pre-pubertal mammary gland, where it may mark the earliest prospective luminal precursor cells. Pertinently, CD55 marks the functional separation of the megakaryocytic-erythroid and lympho-myeloid branches in the haematopoietic system^18^. In pubertal glands, a tight cluster of cells that co-express Cd55 with *Aldh1a3*, a marker of mammary progenitor cells^4, 7, 49^ and the receptor tyrosine kinase *Axl* that has been recently implicated in the epithelial mesenchymal transition^50^, could be distinguished. In adult tissue, CD55 was found to mark progenitor cells exclusively restricted to the luminal lineage. Moreover, the CD55^+^ subset falls within the CD14^+^ (and CD61^+^) luminal fractions, where it substantially enriches for highly clonogenic progenitor cells. Interestingly, *Axin-2* cell-fate mapping studies have indicated that the output of cells induced at different stages can differ markedly^51^. Similar observations may apply to CD55^+^ cells during mammary gland development. Although CD55 has been implicated in breast tumorigenesis^52^, its precise role remains to be determined.

Notably, MaSCs were not readily definable through scRNA-seq analyses. Conversely, functional studies based on cell surface marker expression and flow cytometry have led to further fractionation of stem cells from the basal compartment. For example, EpCAM and Procr enrich for MaSCs in combination with other markers^7, 53^. More recently, *Lgr5* and Tspan8 were utilized to resolve three basal subsets with stem cell activity, one of which was highly enriched for dormant MaSCs^26^. Whilst molecular profiling represents a powerful tool for unraveling heterogeneity on a global scale, an understanding of the molecular and functional diversity amongst stem cells in the basal compartment may only be possible through fractionation based on cell surface markers. Interestingly, although hematopoietic stem cells (HSCs) are well-defined, scRNA-seq analysis of human hematopoietic cells indicated a continuum of molecular states downstream of HSCs, implying that lineage restriction can occur in multiple directions^54^.

In summary, the deconstruction of heterogeneity at different developmental time-points by ordering individual cells on the basis of transcriptome similarity has led to unexpected findings on the transcriptional landscape of mammary epithelial cells. The gene expression programs of the definitive mammary lineages appear to be defined near the onset of puberty, with the majority of epithelial cells existing in a unique molecular state prior to this period. In the adult mammary gland, at least two novel intermediate subsets were delineated. The lineage-primed subset evident amongst purified adult basal cells by hierarchical clustering suggests that a basal expression state precedes commitment to the luminal lineage. Collectively, these data on the molecular identity of individual mammary epithelial cells should provide a useful resource for future studies on understanding the molecular networks that drive specification and differentiation.

## Methods

### Mouse strains

The FVB/NJ mouse strain was used for preparation of mammary epithelial cell suspensions for all scRNA-seq and RT-PCR work. All animal experiments conformed to regulatory standards and were approved by the Walter and Eliza Hall Institute Animal Ethics Committee.

### Mammary cell preparation and cell sorting

Mammary glands from 2-week-old (precisely 14 days old), 5-week-old (between 4.7–5 weeks and designated 5 weeks herein) and 10-week-old female mice were collected and single-cell suspensions were prepared and stained^45^. The following antibodies were used: FITC anti-mouse CD29 (rat, clone HMβ1-1, BioLegend Cat#102206, 1/200 dilution), Pacific Blue anti-mouse CD24 (Armenian Hamster, clone M1/69, BioLegend Cat#101820, 1/200), APC anti-mouse CD31 (rat, clone 390, BioLegend Cat#102410, 1/40 dilution), APC anti-mouse CD45 (rat, clone 30-F-11, BioLegend Cat#103112, 1/100 dilution), APC anti-mouse TER-119/erythroid cell (rat, clone TER-119, Biolegend Cat#116212, 1/100 dilution), PE anti-mouse CD55 (Armenian hamster, clone RIKO-3, BioLegend #131803, 1/50 dilution), and biotin anti-mouse CD14 (rat, clone Sa2–8, eBioscience #13-0141-85, 1/100 dilution). To exclude dead cells, cells were re-suspended in 0.5 μg/ml propidium iodide prior to analysis. FACS analysis and cell sorting were performed on a FACS Aria (Becton Dickinson). The Lin^–^ population was defined as Ter119^–^CD31^−^ CD45^–^. FACS data were analyzed using FlowJo software (v 10.1r7, Tree Star).

### Colony-forming assays

For colony-forming assays, freshly sorted cells were embedded in Matrigel (BD PharMingen) and cultured in DMEM/F12 medium supplemented with 1 mM glutamine, 5 μg/ml insulin, 500 ng/ml hydrocortisone, 10 ng/ml epidermal growth factor, 20 ng/ml cholera toxin, and 5% FCS, in a low-oxygen incubator at 37 °C for 7–8 days. Images were captured and colony number was evaluated by ImageJ.

### Single cell capture and library preparation for sequencing

Freshly sorted epithelial cells were submitted to the Fluidigm C1 machine for single-cell capture and complementary DNA (cDNA) preparation according to the manufacturer’s specifications. The 10–15 μm C1 integrated fluidics circuits (IFCs) were used to capture cells, convert polyA^+^ RNA into cDNA, and perform cDNA amplification. Cells were visualized under the microscope to ensure integrity of the captured single cells prior to cDNA preparation. For two experiments, single-cell capture was confirmed by examining 3D images of 100's of individual cells through acquiring Z-stacks of mammary epithelial cells captured on C1 IFCs. The 3D analysis corroborated observations made by regular microscopy. The libraries were prepared using the Nextera XT kit and sequencing was carried out on either an Illumina HiSeq 2000 to achieve 100 bp paired-end reads on a NextSeq 500 to achieve paired-end 75 bp reads.

For high through-put single-cell studies, a 10X Genomics Chromium machine was used for 3000–5000 single-cell capture and cDNA preparation according to the Single Cell 3′ Protocol recommended by the manufacturer. The cells are first washed and prepared in an ideal concentration. The machine partitions thousands of cells into nanoliter-scale Gel Bead-In-EMulsions (GEMs), where all generated cDNA share a common 10x Genomics Barcode but uses a pool of ~750,000 barcodes to separately index each cell’s transcriptome. The silane magnetic beads and Solid Phase Reversible Immobilization beads were used to clean up the GEM reaction mixture and the barcoded cDNA was then amplified in a PCR step. Optimal cDNA amplicon size was achieved using Covaris machine prior to library construction. The P7 and R2 primers were added during the GEM incubation and the P5, and R1 during library construction via end repair, A-tailing, adapter ligation and PCR. The final libraries contain the P5 and P7 primers used in Illumina bridge amplification. Sequencing was carried out on an Illumina Nextseq 500 to achieve 75 bp reads.

### Multiplex single-cell RT-PCR

Single-cell RT-PCR were performed using 96:96 IFC microfluidic chips (Fluidigm). Single cells were flow sorted directly into the individual wells of 96-well PCR plates containing 5 μl/well of CellsDirect PCR mix and RNase Inhibitor mix. Following single-cell sorting, each well was supplemented with 1 μl SuperScript III RT/Platinum Taq (Invitrogen), 1.5 μl Tris-EDTA (TE) buffer and 2.5 μl of 96 pooled TaqMan assays (Applied Biosystems). Single-cell lysates were directly reverse transcribed into cDNA (50 °C for 15 min, 95 °C for 2 min) and pre-amplified for 14 PCR cycles (60 °C for 4 min, 95 °C for 15 s). A mixture containing 2.25 μl amplified cDNA (diluted 1:5), 2.5 μl TaqMan quantitative PCR (qPCR) mix (Applied Biosystems) and 0.25 μl Fluidigm sample loading agent was prepared and loaded onto the IFC chip. A 2.5 μl aliquot of TaqMan assay was mixed with 2.5 μl Fluidigm assay loading agent and loaded onto the IFC chip. The 96:96 chip was then submitted to HX IFC Controller (Fluidigm) before setting up qPCR run on Biomark real-time PCR machine (Fluidigm) following the manufacturer’s instructions; the data were analyzed using the Fluidigm program. Cells not expressing ACTB (β-actin) and GAPDH (Glyceraldehyde 3-phosphate dehydrogenase) were removed from the analysis.

### scRNA-seq bioinformatic analysis

Read pairs from the Fluidigm C1 scRNA-sequencing were mapped to the mm10 mouse genome using the Subread aligner^55^ and assigned to genes using featureCounts^56^ and Rsubread’s built-in refSeq annotation. Mitochondrial genes, ribosomal genes, and Entrez gene IDs without current annotation were removed. To ensure good coverage per cell, cells were dropped from the analysis if fewer than 100,000 read pairs were assigned to the remaining genes or fewer than 1500 genes were detected. For epithelial cells from pregnant mice and basal cells from adult mice, these thresholds were relaxed to 50,000 read pairs and 1000 genes. Finally, genes that failed to achieve 1 count-per-million (CPM) in at least 3 cells were filtered from the analyses. Illumina output from 10X Genomic Chromium sequencing reads was processed using Cell Ranger v2.0. Genewise read counts were exported to Matrix Market format files and read into R.

Statistical analyses of the C1 data were conducted using the edgeR^57^, limma^58^, and Rtsne software packages for R. MDS plots were generated with edgeR’s plotMDS function. Distances on the MDS plots represent leading log fold change, which is the root mean square average of the largest 500 expression log2-fold changes between each pair of cells. Ternary plots were generated using the vcd package with quantile normalized log2-CPM values. Log2-RPKM values calculated using edgeR’s rpkm function with a prior count of 5. Heatmaps were generated using the heatmap.2 function of the gplots package. Log2-RPKM values were standardized to have mean 0 and standard deviation 1 for each gene before producing the heatmaps, after which genes and cells were clustered by Euclidean distance. Differential expression analyses used likelihood ratio tests with negative binomial dispersions estimated by the estimateDisp function^57, 59^. Gene ontology and Kyoto Encyclopedia of Genes and Genomes (KEGG) analyses were performed using the goana and kegga functions. t-SNE plots were generated using the Rtsne package.

### Bulk RNA-seq bioinformatic analysis

Signature genes for the basal and luminal cell lineages were obtained from previously published RNA-seq data^22^. The differential expression analysis was performed using the glmTreat function in edgeR with a fold change threshold of 3. Only protein-coding genes were considered in the analysis. Signatures genes for basal, LP, and ML cell types were obtained from other previously published RNA-seq data^8^. Genes were considered cell type specific if they were upregulated in one cell type vs. both other types. The comparisons were performed with a limma TREAT fold change threshold^60^ of 2 for basal vs LP and basal vs ML and 1.3 for LP vs ML. A false discovery rate (FDR) cut-off of 0.05 was applied for each comparison.

### Protein network analysis

The mouse interactome is sparse compared with that available for human^61^, so mouse genes were mapped to human orthologs using the mouse-human ortholog mapping available through the Mouse Genome Informatics resource^62^. Protein interactions were retrieved, from a large collection of manually curated experimentally identified protein interactions^63^, for the orthologs of genes found to be DE between pre-puberty, puberty and adulthood from the scRNA-seq with FDR < 0.001. Only direct interactions between genes were used. Gene Ontology terms “signal transduction” (GO:0007165) and “TF activity” (GO:0003700) were used to identify signal transduction and transcript factor genes, respectively. Networks were merged with differential expression data and functional categories, and imported to Cytoscape for analysis and visualization. Networks were generated for the 2 vs. 5 week comparison, and for the 5 vs. 10 week comparison. Small subnetworks corresponding to sets of genes of interest were sampled from these background networks.

### Single-cell western analysis

Freshly sorted single-cell suspensions (30,000 cells per ml) were loaded onto the scWest chip for 20 min. Optical visualization was performed to confirm and mark the single-cell capture sites followed by gentle washing in resuspension buffer according to the manufacturer’s conditions. The scWest chip was then submitted to the Milo machine (Protein Simple) for lysis, electrophoresis and protein immobilization. Protein targets were probed on-chip with primary antibodies against Keratin 14 (rabbit polyclonal, Thermofisher, cat#: LBVRB-9020-P0, 1:80 dilution) and Keratin 8/18 (rat, clone: TROMA-1, DSHB, 1:80 dilution) overnight at 4 °C and then with secondary antibodies, either anti-rat Alexa-647 (Invitrogen; 1:100 dilution) or anti-rabbit Alexa-555 (Invitrogen; 1:100 dilution) for one hour at room temperature. The probed chip was washed, air-dried and analyzed using a fluorescence microarray scanner (Innoscan 710).

### Confocal analysis on whole-mounts and histological sections of mammary glands

Tissues were dissected, fixed in 4% paraformaldehyde and incubated overnight at 4 °C with primary antibodies. The following day, tissues were incubated with secondary antibodies. Tissues were subsequently incubated in 80% glycerol before dissection for three-dimensional imaging^64^. The following primary antibodies were used: Keratin 14 (rabbit polyclonal, Thermofisher, cat#: LBVRB-9020-P0, 1:500 dilution), Keratin 8/K18 (rat, clone: TROMA-I, DSHB, 1:500 dilution), and Vimentin (mouse, clone LN-6, Sigma cat# V2258, 1:200 dilution). All secondary antibodies were Alexa Fluor-conjugated: anti-rabbit Alexa Fluor 488 (Invitrogen; 1:500 dilution), anti-mouse Alexa Fluor 555 (Invitrogen; 1:500 dilution), anti-rat Alexa Fluor 647 (Invitrogen; 1:500 dilution).

### Data availability

Sequencing data have been deposited in the GEO database under accession codes: GSE98131 (C1 data) and GSE103275 (10X data). The authors declare that all data supporting the findings of this study are available within the article and its Supplementary Information files.

## Electronic supplementary material

Supplementary Information
