# Supplementary material for: Construction of developmental lineage relationships in the mouse mammary gland by single-cell RNA profiling
Source: Nat Commun. 2017 Nov 20;8:1627. doi: 10.1038/s41467-017-01560-x (PMC5696379; doi:10.1038/s41467-017-01560-x)
Supplement: Supplementary file 1 — Supplementary Information [file 41467_2017_1560_MOESM1_ESM.pdf]

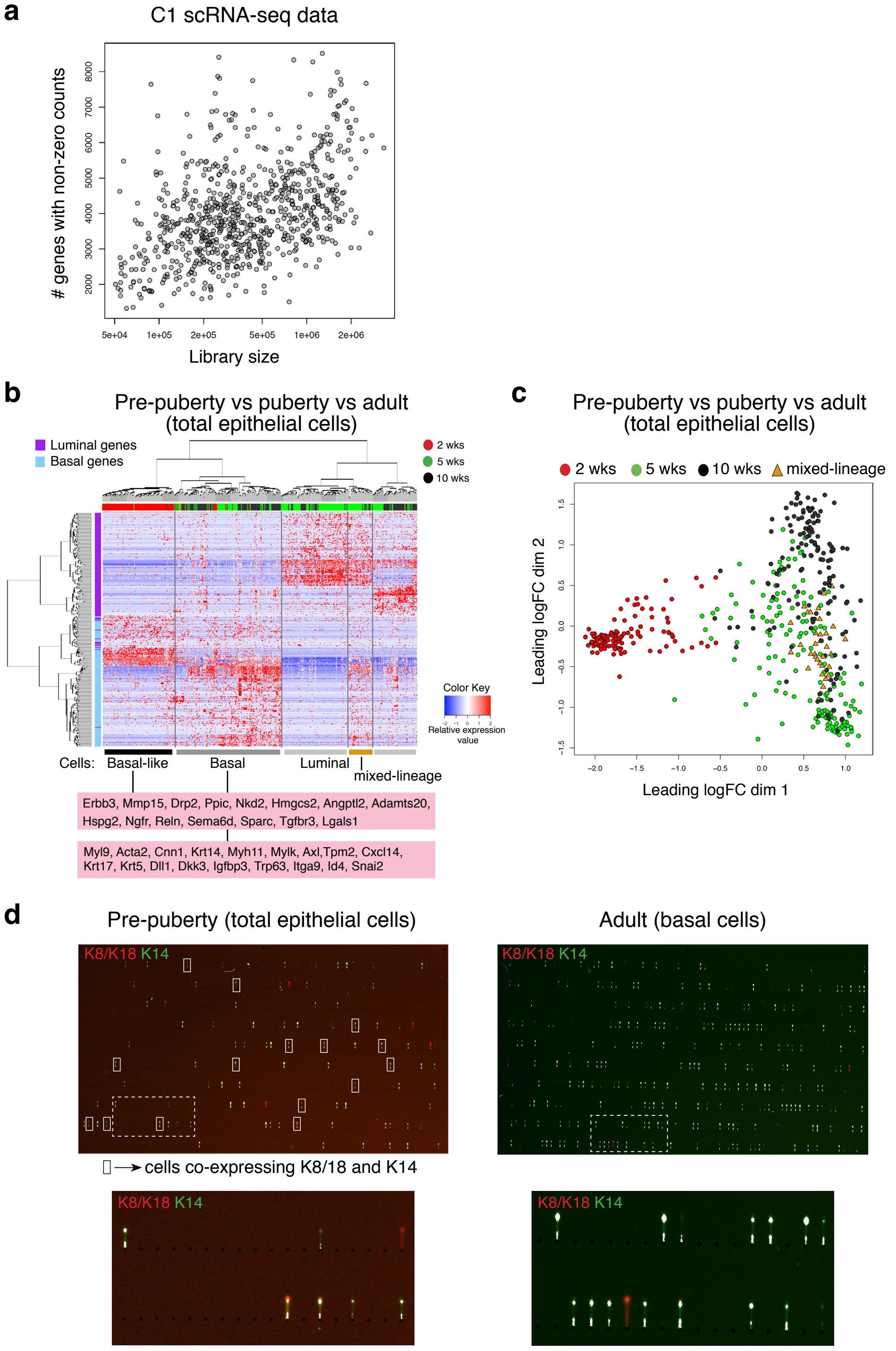

Supplementary Figure 1. Single-cell transcriptome analysis across three developmental stages. (a) Scatterplot showing the total number of reads (x-axis) and the number of expressed genes (y-axis) for all cells profiled using the Fluidigm C1 platform. **(b)** Expression heatmap of epithelial cells from pre-pubertal (2 weeks; red), pubertal (5 weeks; green) and adult (10 weeks; black) mice (same cells as depicted in Fig. 1b-c). Rows correspond to genes known to be basal or luminal- specific[1] (200 genes for each lineage). Genes expressed in fewer than 5% of cells were excluded. Five major cell clusters are marked. Red = high expression; Blue = low expression. **(c)** Multi-dimensional scaling plot of the same cells as in Fig. 1b-c, with cells from the mixed-lineage cluster in (b) marked as yellow triangles. The plot is the same as in Fig. 1b except for marking of mixed-lineage cells. **(d)** Single-cell westerns of single total epithelial cells from pubertal (n=6 mice) and single basal cells from adult mammary glands (n=4 mice) using the Milo single-cell western system. The scWest chips were probed with anti-Keratin 8/18 (luminal marker) and anti-Keratin 14 (basal marker) antibodies. Top panels show expression analysis for 118 pre-pubertal epithelial cells (left) and 220 adult basal cells (right). Fifteen pre-pubertal epithelial cells (indicated by rectangles) showed strong co-staining for K8/18 and K14. Magnified images (dashed rectangles) are shown in the lower panels.

a Transcription factor and signal transducer network (2 vs 5 wks)

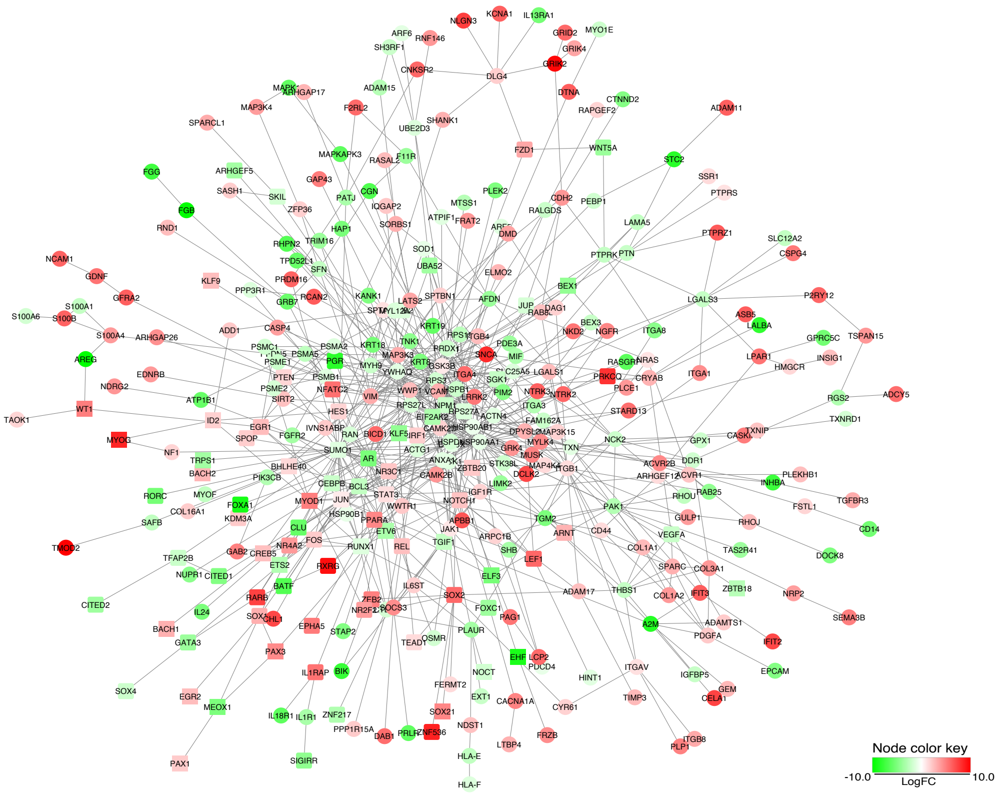

b Transcription factor and signal transducer network (5 vs 10 wks)

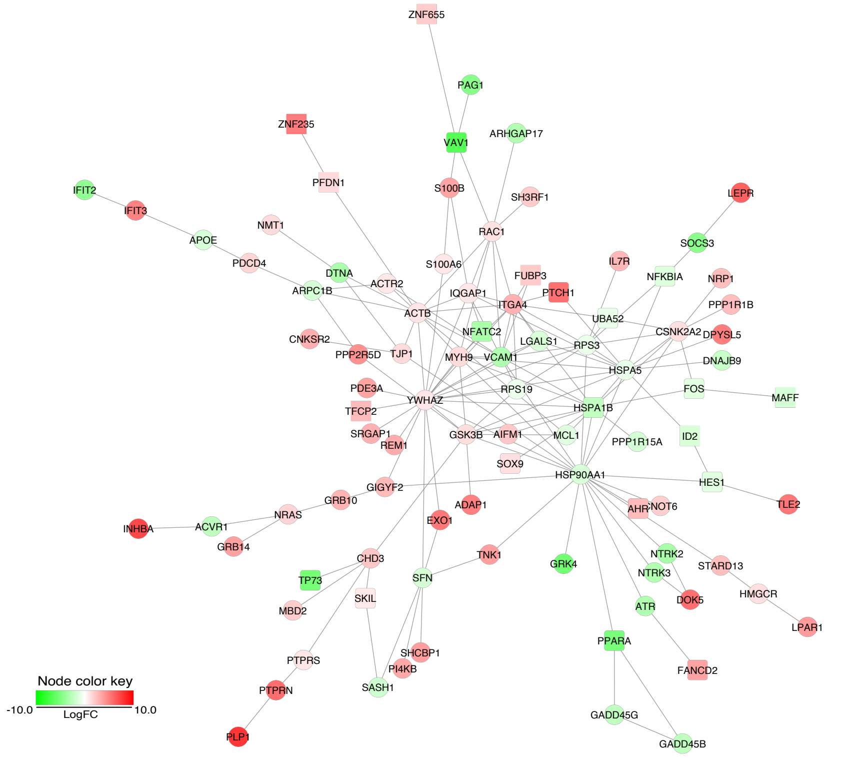

Supplementary Figure 2. Developmental stages are marked by unique protein-protein interactions. (a) Protein interaction network of transcription factors and signal transducers that are up-regulated (red) or down-regulated (green) in pre-pubertal cells vs pubertal cells. Node shape represents protein function (circle = signal transduction; square = transcription factor; rounded square = dual function and diamond = none of the above). **(b)** Protein interaction network of transcription factors and signal transducers that are up-regulated (red) or down-regulated (green) in pubertal vs adult cells.

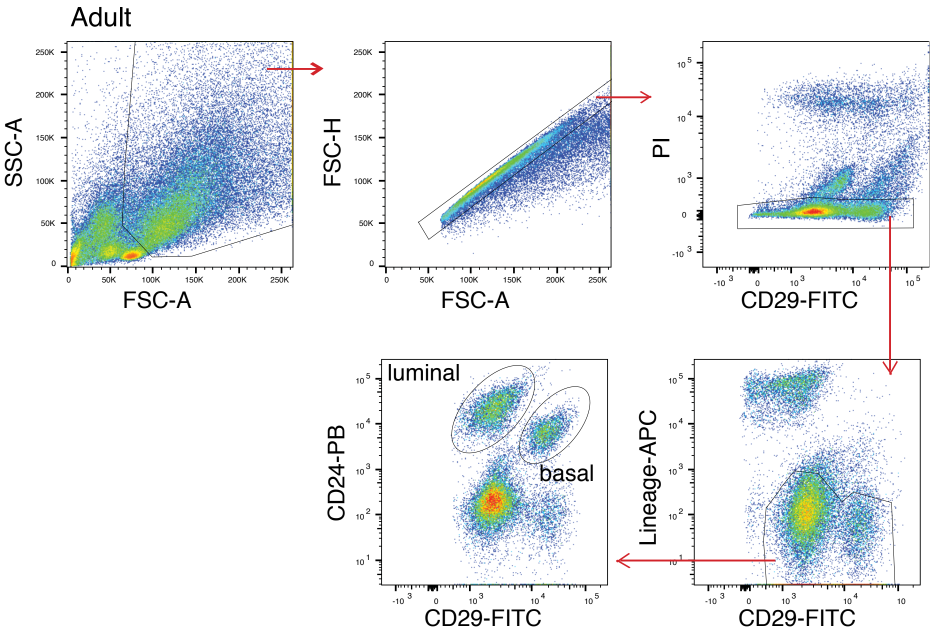

Supplementary Figure 3. Flow cytometry of mammary epithelial cell preparations. Representative flow cytometric sorting strategy for the isolation of $Lin^{-}CD29^{hi}CD24^{+}$ (basal) and $Lin^{-}CD29^{lo}CD24^{+}$ (luminal) mammary epithelial cells from 9-10 week-old mice. Lineage negative cells are $CD31^{-}CD45^{-}TER119^{-}$ (n>10 independent experiments, 4 mice per experiment).

a

Cluster 5 genes

Col3a1, Fgl2, Acta1, Ly6a, Gsn, Igfbp7, Gpx3, Rarres2, Ly6c1, Sparcl1, Igfbp6, Crip1, Anxa1, Aldoa, Cpe, Sncg, Gdpd3, Cd34, Egfl7, Prnp, Rasd1, Nrarp, Qpct, Cd55, Lgals3, Cystm1, Sfrp2, Ugdh, Id3, Serping1, Eno3

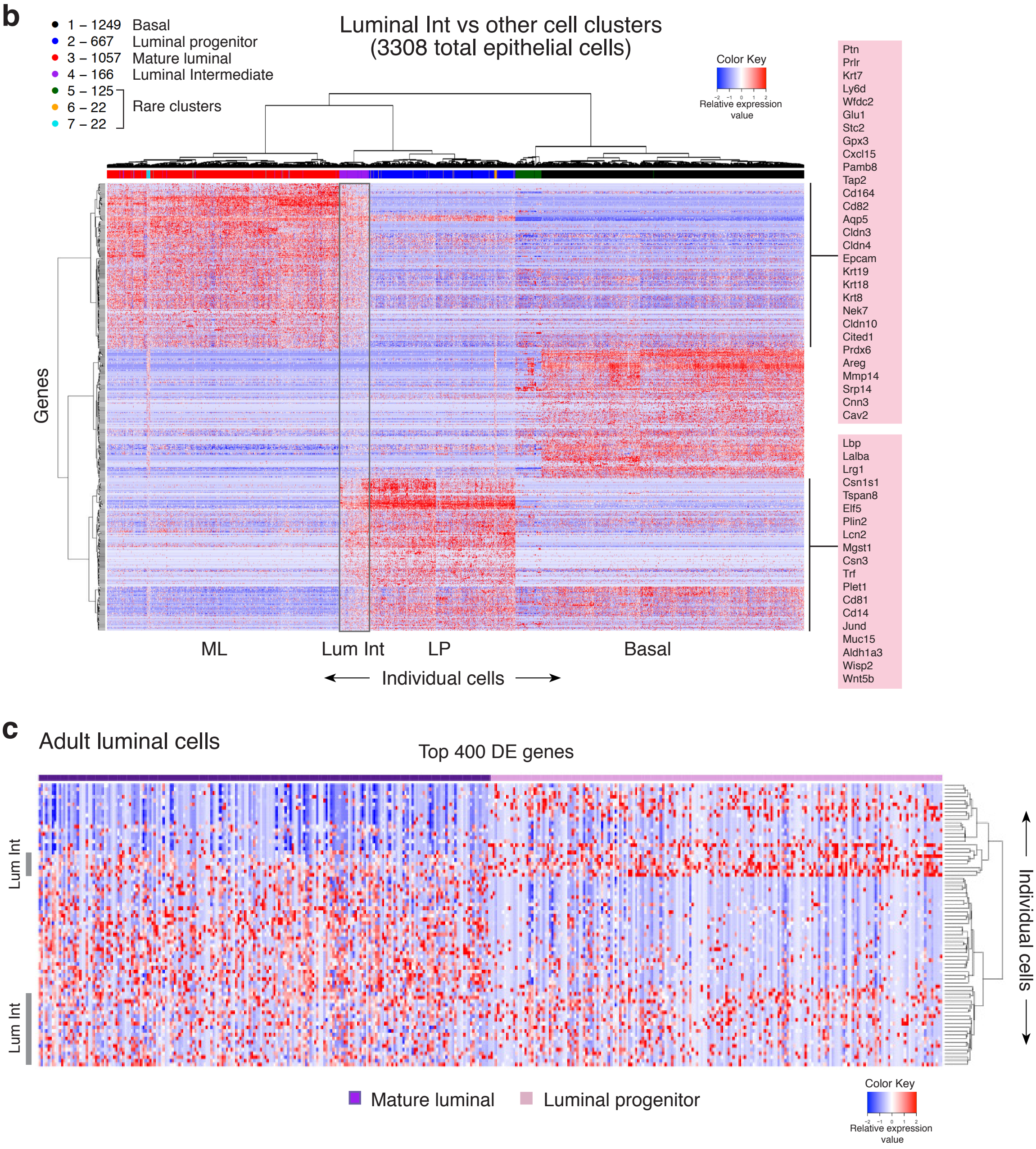

Supplementary Figure 4. Heterogeneity amongst purified luminal cells in the adult mammary gland. **(a)** Most highly expressed genes in Cluster 5 of the t-SNE plot shown in Fig. 4a, demonstrating basal character. **(b)** Expression heatmap of 3308 adult total epithelial cells (as in Fig. 4). Cells are colored according to t-SNE cell clusters from Fig. 4a. Rows correspond to 553 genes chosen to illustrate the expression profiles of clusters 4-7. Genes have intermediate expression in Lum Int vs LP and ML, or intermediate expression in cluster 6 vs basal and LP, or intermediate expression in cluster 7 vs basal and ML, or intermediate expression in cluster 5 vs basal and the luminal clusters, or else are up-regulated in basal compared to the luminal clusters. Boxes (right side) encompass selected genes shared between Lum Int and ML, and Lum Int and LP. **(c)** Expression heatmap of purified (sorted) luminal cells (76 cells) from adult mammary glands (n=4 mice) generated using the Fluidigm C1 system, based on expression of the top DE genes between luminal progenitor (LP) and mature luminal cells (ML)[2,3] (200 LP and 200 ML-specific genes). Hierarchical clustering separates LP cells (upper cluster) from ML cells (lower cluster). Second level clusters delineate luminal intermediate cell populations (grey bars) within each of the major subsets.

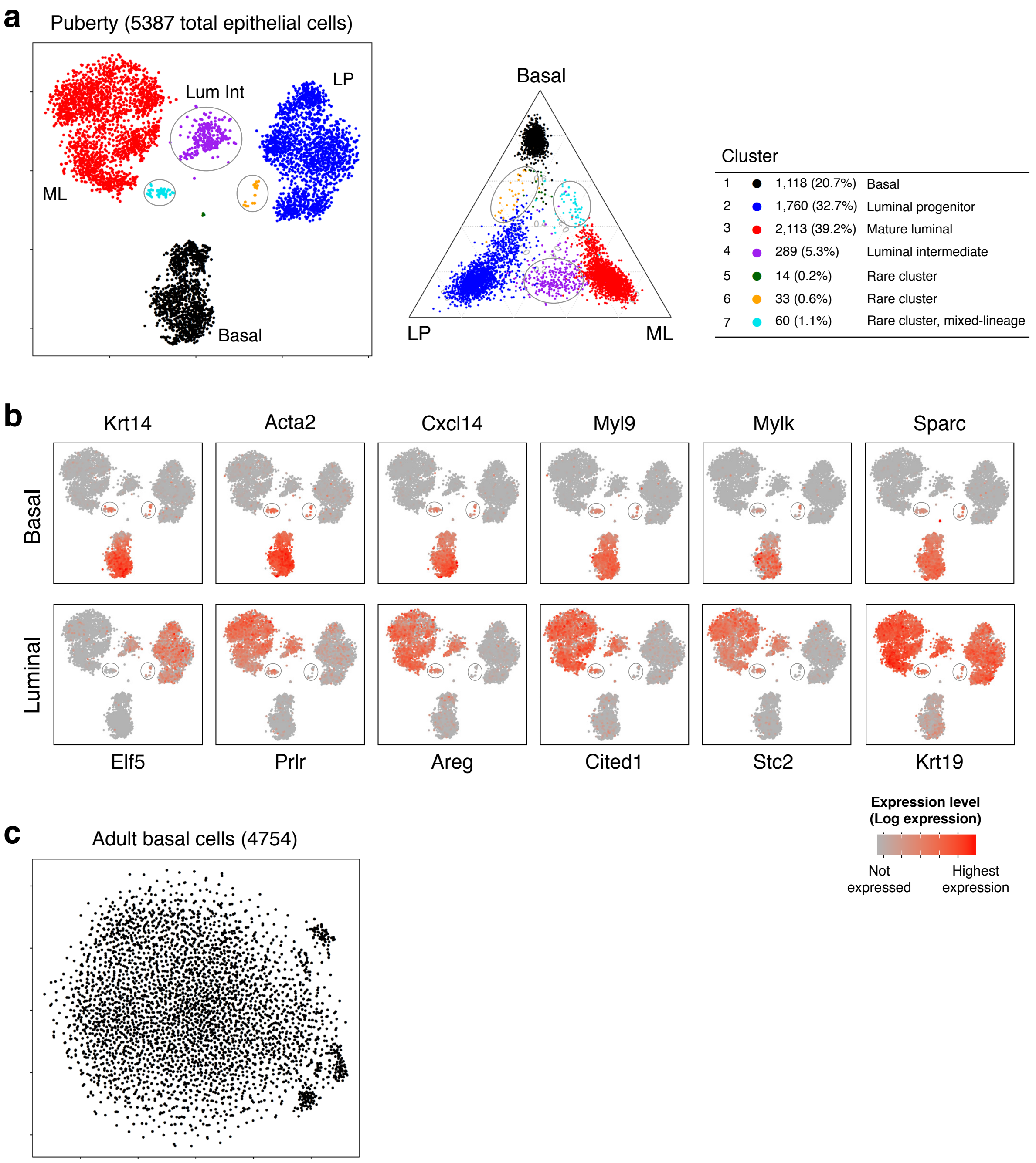

Supplementary Figure 5. Single-cell RNA-seq of cells from pubertal mammary glands reveals the presence of luminal intermediate and mixed-lineage subsets. **(a)** t-SNE plot (left panel) of the transcriptomes of total epithelial cells from pubertal mammary glands (n=6 mice) generated using the 10X Genomics Chromium platform. The major clusters could be identified as basal (cluster 1), luminal progenitor (LP; cluster 2), mature luminal (ML; cluster 3), luminal intermediate (Lum Int; cluster 4) and cluster 7 (mixed-lineage in sky blue, 60 cells), as indicated. The t-SNE clusters were overlaid onto a ternary plot (right panel) showing the expression profile of each cell relative to the basal, LP and ML lineages. Cells are plotted according to the proportion of genes they express that are specific to the three cell types. **(b)** The epithelial cell clusters were interrogated for the expression of typical basal and luminal genes to determine their patterning amongst the cell clusters. Color indicates expression level in log2-CPM. **(c)** t-SNE plot of the transcriptomes of 4754 purified adult basal cells (n=4 adult mice) generated using the 10X Genomics Chromium platform. A largely homogeneous population was observed, consistent with data presented in Fig. 4a.

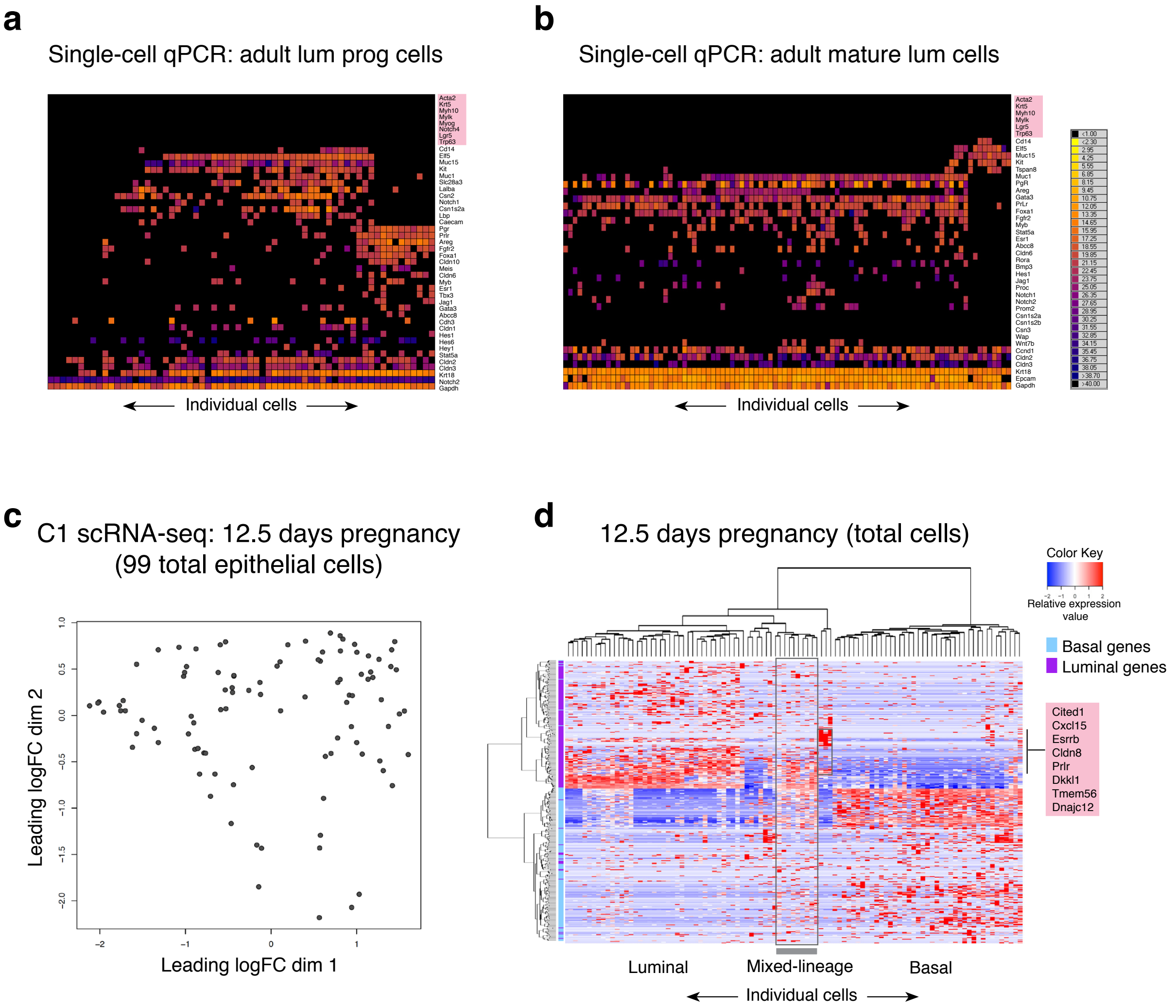

Supplementary Figure 6. Single-cell RNA expression analysis of adult luminal cells and total epithelial cells from pregnancy. Heatmaps showing qRT-PCR expression analysis for 64 sorted adult luminal progenitor **(a)** and 94 mature luminal cells **(b)**; n= 6 mice per sort, representative of 2 or 3 experiments for mature and luminal progenitor cells, respectively. Taqman assays for 8 basal and 36 luminal lineage genes using the Fluidigm Biomark multiplexed qPCR platform are shown, including the house-keeping gene Gapdh. The color key shows Ct values from black (Ct > 40, no expression) to yellow (highest expression). **(c)** Multi-dimensional scaling plot of 99 mid-pregnancy (12.5 days) epithelial cells (n=2 mice) showing gene expression changes. Distances on the plot correspond to leading log2 fold-change between any pair of cells. **(d)** Heatmap showing hierarchical clustering of 99 mid-pregnancy (12.5 days) epithelial cells based on expression of the top 200 basal and top 200 luminal lineage genes[1,2] (Red = high expression; Blue = low expression). A substantial mixed-lineage cluster that expresses multiple core basal and luminal lineage genes, and a tight cluster highly enriched for luminal genes, are indicated.

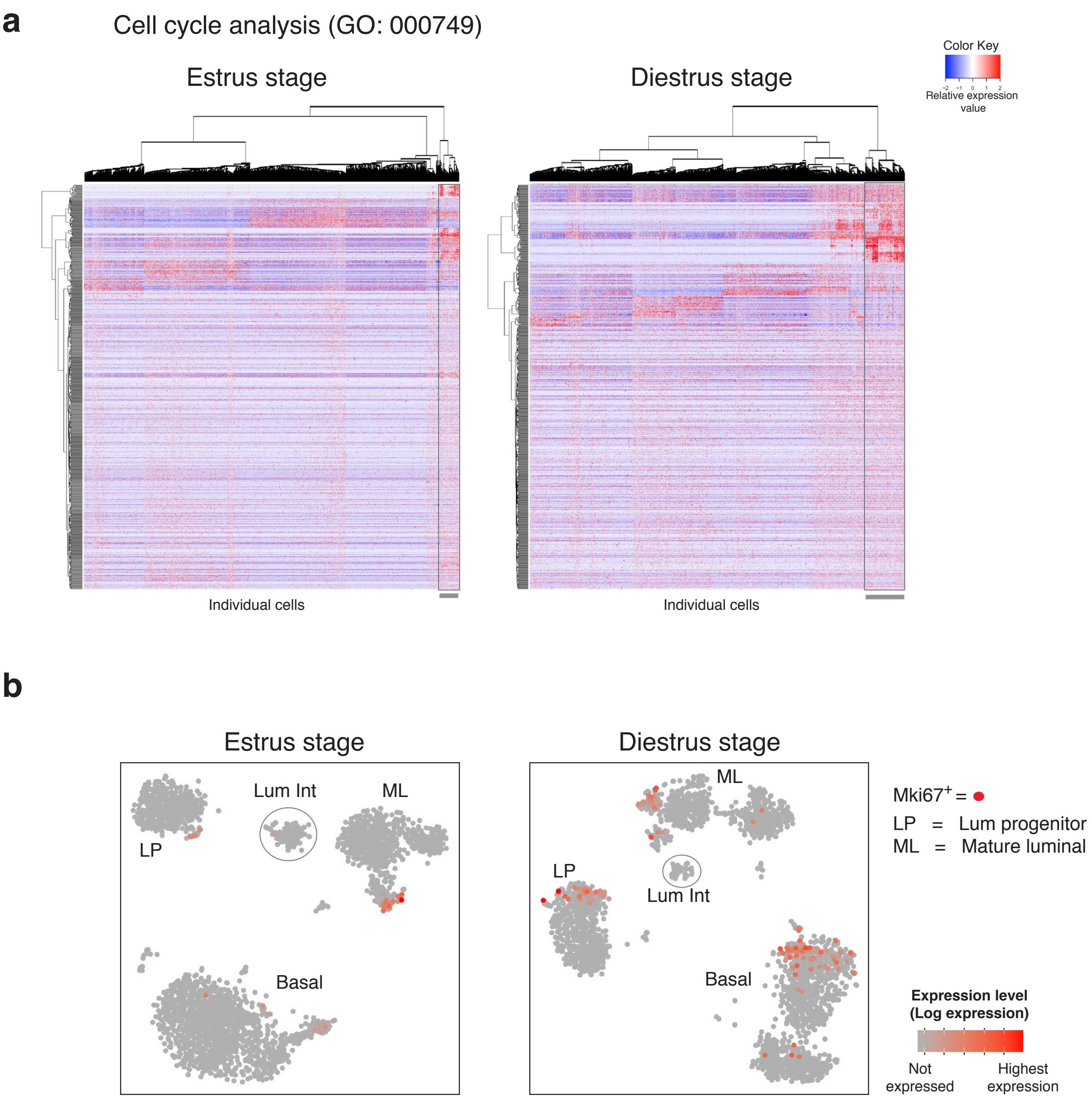

Supplementary Figure 7. Heatmaps of adult total epithelial cells at Estrus and Diestrus stages by expression of cell cycle genes. (a) Columns correspond to the same cells as in Fig. 6. Rows correspond to genes associated with the Cell Cycle gene ontology term GO:000749; 920 genes are shown for estrus and 961 genes for diestrus. Hierarchical clustering identifies a cluster of cells on the right in each heatmap with high expression of key cell cycle genes. Cells in this cluster are marked red in Fig. 6b. **(b)** t-SNE plots showing expression of Mki67 (Ki67) in the cell clusters at estrus and diestrus.

Supplementary Table 1. Summary of single cell RNA-seq experiments

		Number of cells†	
**Stage**	**Cell lineage**	**C1 Fluidigm**	**10X Chromium**
Pre-puberty (2 wks)	Total epithelium	117	–
Puberty (5 wks)	Total epithelium	181	5387
Adult (10 wks)	Total epithelium	162	3308
Pregnancy (12.5 days)	Total epithelium	99	–
Estrus	Total epithelium	–	2729
Diestrus	Total epithelium	–	2439
Adult (10 wks)	Basal cells	145	4754
Adult (10 wks)	Luminal cells	76	–

† The table gives the number of cells that were retained in the analyses after filtering out cells with low sequencing coverage. Columns correspond to developmental stage, cell lineage and scRNA-seq platform.

Supplementary Table 2. Gene clusters defined by unsupervised clustering of single cell RNA-seq data across developmental stages

Gene Clusters†					
I	II	III	IV	V	VI
Sparc	Prdm16	Pcnp	Slitrk5	Cldn7	Csn2
Tgfbr3	Rxrg	Hmgcr	Dclk2	Cldn4	Csn1s2a
Lgals1	Megf9	Nf1	Rftn2	Epcam	Csn1s1
Sorbs1	Hmgcs2	Pten	Nfasc	Krt8	Cebpb
Timp3	Lats2	Dip2b	Soga3	Krt18	Ehf
Scn7a	Sostdc1	Sptbn1	Iffo1	Ly6e	Cnn1
Vim	Plekha4	Birc6	Plxnb3	Sox9	Krt14
Cadm1	Npr3	Sept2	Cdh20	Gata3	Myh11
	Ank3	Gsk3b	Epha5	Dsg2	Serpinb11
	Nr2f2	Nras	Pax3	Areg	Nrg1
	Mpz	Gpc1	Ntrk3	Prlr	Ctnnd2
	Mbp	Myef2	Foxd3	Krt19	Eno1
	Fbxo7	Ptprs	Mmd2	Krt7	Wbp5
	Sox6	Actb	Tspan18	Cd164	Prdx1
	Igsf11		Foxp2	Ly6d	Mir682
	Spry2		Ptprd	Ptn	Dusp7
	Meg3		Dlx2	Foxa1	Serpinb5
	Bach1		Ncam2	Fgfr2	Calm2
	Dbi		Lef1	Wnt5a	Cd24a
	Azin1		Itga4	Kif5c	Pabpc1
	Cd59a		Adamts20	Pgr	Sub1
	Tiparp		Itga1	Cxcl15	Ptma
	Casp4		Ldb2	Klf5	Npm1
	Col5a1		Adam19	Prom2	
	Lama2		Mcam	Il33	
	Col1a2		Sfrp1	Inhba	
	Lpar1		Nkd2	Myh9	
	Nrn1		Slitrk6	Cbx3	
	Dmd		Ptprz1	Cdkn2b	
	Prnp		Fabp7		
	Hspg2		Cdh2		
	Stard13		Slitrk2		
	Ncam1		Itgb4		
	Zeb2		Hes1		
	Cdh19		Egr1		
	Plp1		Fos		
	Matn2		Sox2		
	Prex2		Adam11		
	Nid2				
	Fgl2				
	L1cam				

† These gene clusters refer to those highlighted on the y-axis in Fig. 1c; examples of highly expressed genes are listed.

Supplementary Table 3. Top differentially expressed (DE) genes in adult basal and luminal cells

Basal†					Luminal				
Wif1	Chst11	1500015O10Rik	Tpst1	Trim29	Stc2	Tph1	Liph	Pdzk1ip1	Igfals
Antxr1	Tgfbr3	Fmod	Klhl29	Adamts1	Hp	Csn3	Gdpd1	Cmtm8	Folr1
Cpxm2	Hdgfrp3	Scn7a	Nebl	Serpinf1	Cited1	Cadps2	Areg	Btc	Cadm4
Cacna2d1	Mrvi1	Sparc	Nipal4	Cntn2	Rasef	Nectin4	Slc1a1	Tmem30b	Stra6l
Krt5	Rcn3	Cttnbp2	Fbn1	Igsf10	Rhov	Esm1	Csn1s1	Manba	Mmp16
Cdh3	Id4	Psd2	Col5a2	Pla2g7	Prlr	Cel	Foxi1	Myo5c	Faah
Col14a1	Kcnmb1	Tenm2	Ppic	Asxl3	Foxa1	Slc44a4	Gpd1	Bik	Ugt8a
Dll1	Bgn	Dpysl3	Ngfr	Kif1a	Ly6d	Rassf6	Cldn4	Dnajc6	Glycam1
Col17a1	Acta2	Lrrc8c	Clmp	Aff3	Muc4	Gipc2	Klk10	Lrrc8b	Hepacam2
Slit3	Rnf150	Rtn1	Cdh13	Cpne8	Ocln	Krt18	R3hdml	Lgals3	Cxcl15
Fam129a	Itga9	Wnt6	Aoc3	Gm21190	Arg1	Fam25c	Msx2	Ceacam1	S100a8
Axl	Tmem200a	Asb2	Tceal3	Pknox2	2010300C02Rik	Wfdc18	Nupr1	Unc13b	Cd14
Col5a1	Rnase4	Sobp	Lrp1	P3h1	Slc28a3	Sulf2	Atp1b1	Cecr2	Gabrp
Tgfb1i1	Pcdh7	Ckb	Palm	Ptch2	Marveld2	Ctsh	Tmprss13	Enpp3	Agtr1a
Elovl4	Bzrap1	Mark1	Wnt11	Phyhip	Pkp2	Ppp1r9a	Capn8	Cd55	Tacstd2
Chst15	Col7a1	Spon2	Dsc3	Efcab1	Nav3	Dock8	Crym	Bdh1	Hhipl2
Tpm2	Irx4	Col4a1	Abcb1b	Nkd2	Cldn3	Lcp1	Ano3	Trf	Cntnap2
Postn	Fam65b	Msrb3	Scn5a	Fjx1	Bglap3	Tmem56	Ehf	Cldn1	Kcnn4
Hmcn1	Slc2a4	Cxcl14	Nckap5l	Matn4	Cgn	Plac8	Atp6v1b1	Nxf7	Clcn3
Igfbp3	Krt17	Eml1	Begain	C1s1	Btn1a1	Bcl2l14	Erbb3	Igsf5	Lalba
Vwa1	Cacna1c	Ism1	Nrg2	Fat2	Spp1	Aqp5	Cldn6	Fer1l4	Nup210
Aebp1	Snai2	Nrg1	Adcy7	Sema6a	Vill	Ptn	Spns2	Slc46a3	Plb1
Mylk	Pdpn	Nectin3	Galnt16	Eya1	Krt19	Tnfaip2	Itih2	Cystm1	Ncald
Angptl2	Zfp365	Gja1	Wnt10a	Hunk	Gca	Csn1s2a	Clu	Csn2	Arhgef38
Sdk2	Tspan18	Gas1	Cacna1g	Adamts2	Elf5	Thsd4	Cldn7	Tmc4	Hmgcs2
Igfbp2	Myh11	Mef2c	Dact3	Gm17019	Clic6	2210407C18Rik	AA986860	Upk2	Rspo1
Cnn1	Lhfp	Jph2	Ephb1	Ttc7b	Sort1	Esrrb	Slc22a18	Rftn1	Fam174b
Jag2	Loxl3	Htr1d	Lgals1	Serpinb10	Shank2	Wfdc3	Scnn1a	Mmp15	Trim6
Moxd1	Gfra1	Sema6d	Serpinb11	Hspg2	Fgb	Tmprss4	Cldn8	Gde1	Adra1a
Vcan	Nptx1	Fbln1	Mapt	Kirrel	Piezo2	Rab11fip1	Trp53inp1	Muc15	Reg3g
Tshz2	Popdc2	Palld	Gm21149	Adamts18	Tspan1	Sptbn2	Klhdc7a	Fcgbp	Aldh1a3
Col4a2	Rai2	Nt5e	Synpo2	Fkbp10	Slc5a1	Bspry	Fam234b	Ltk	Slc45a3
Trp63	Tagln	Hgf	Dkk3	Them5	Mboat1	Ttc39a	Cd82	Wap	Srcin1
Nkd1	Camk4	Drp2	Reln	Ptprm	Ptpn18	Tjp3	Aldoc	Ogfrl1	Pls1
Fstl1	Hs6st2	Soga1	Krt14	Eogt	Smim22	Tmc5	Rasgrf1	Aifm3	Abca3
Col18a1	Kcnq5	Abat	Tril	Speer4d	Zfp750	Muc20	Wnt4	Syne4	Sptlc3
Lmo1	Lama1	Tenm3	Sh3rf3	Rgl1	Mlph	Capn5	Acsbg1	Muc1	2610528J11Rik
Actg2	Plod2	Dchs1	Aatk	Zfhx4	Dkkl1	Ceacam10	Mal2	Slc8b1	Tmem125
Nexn	Adamts20	St3gal2	Dlk2	Bmp7	Tmprss2	Tspan33	Kit	Dnajc12	Prex1
Myl9	Clip3	Ntng2	Spock2	Vwa2	Podxl	Basp1	Abcc8	Rab17	Upk3a

† Basal cells ($Lin^{-}CD29^{hi}CD24^{+}$) and luminal cells ($Lin^{-}CD29^{lo}CD24^{+}$): the signature genes (top 200 DE genes for each lineage) are from Fu *et al.* (2015)[1] (population level, listed in order from top left to bottom right). In the case where a gene was not expressed in a given scRNA-seq dataset, then the next DE gene was included.

Supplementary Table 4. Gene clusters defined by clustering of single-cell RNA-seq data for pubertal and adult mammary glands

Cluster	Gene List †
I	Plac8, Arg1, Foxa1, Gipc2, Stc2, Cxcl15, Ptn, Ly6d, Tmem56, Prlr, Areg, Crym, Cited1, Upk3a, Fgb, Itih2, Dnajc12, Tspan1, Gpd1, Cadps2, Mal2, Cystm1, Gdpd1, Cd82, Lgals3, Tacstd2, Clu, Cldn4, Cldn7, Krt19, Krt18
II	Cd14, Aldoc, Muc1, Bglap3, Muc15, Ano3, Plb1, Foxi1, Muc4, Kit, Kcnn4, Csn1s1, Csn2, Csn1s2a, Ehf, Csn3, Wfdc18, Trf, Clic6, Btn1a1, Lalba, Fcgbp, Spp1
III	Fstl1, Sparc, Tgfbr3, Col14a1, Lgals1, Antxr1, Axl, Fbn1, Palm, St3gal2, Aldh1a3, Cd55, Adamts2, Lrp1, Col5a1
IV	Tpm2, Myl9, Myh11, Cnn1, Krt14, Tagln, Acta2, Mylk, Krt5, Cxcl14, Krt17, Cpne8, Palld, Scn7a, Postn, Gja1, Dpysl3, Pdpn, Fstl1, Sparc, Tgfbr3, Col14a1, Lgals1, Antxr1, Axl, Fbn1
V	Plac8, Arg1, Foxa1, Gipc2, Stc2, Cxcl15, Ptn, Ly6d, Tmem56, Prlr, Areg, Crym, Cited1, Upk3a, Fgb, Itih2, Dnajc12, Tspan1, Gpd1, Cadps2, Mal2, Cystm1, Gdpd1, Cd82, Lgals3, Tacstd2, Clu, Cldn4, Cldn7, Krt19, Krt18, Tpm2, Myl9, Myh11, Cnn1, Krt14, Tagln, Acta2, Mylk, Krt5, Cxcl14, Krt17, Cpne8, Palld, Scn7a, Postn, Gja1, Dpysl3, Pdpn, Fstl1, Sparc, Tgfbr3, Col14a1, Lgals1, Antxr1, Axl, Fbn1
VI	Tpm2, Myl9, Myh11, Cnn1, Krt14, Tagln, Acta2, Mylk, Krt5, Cxcl14, Krt17, Cpne8, Palld, Scn7a, Postn, Gja1, Dpysl3, Pdpn, Fstl1, Sparc, Tgfbr3, Col14a1, Lgals1, Antxr1, Axl, Fbn1, Snai2, Col18a1, Serpinb11, Moxd1, Vcan, Nrg1, Tril, Sema6d, Lama1, Rtn1, Hs6st2, Wif1, Bmp7, Adamts18,<br>Tgfb1i1, Col4a1, Igfbp2
VII	Tpm2, Myl9, Myh11, Cnn1, Krt14, Tagln, Acta2, Mylk, Krt5, Cxcl14, Krt17, Cpne8, Palld, Scn7a, Postn, Mrvi1, Cacna2d1, Tenm2, Synpo2, Gja1, Dpysl3, Pdpn, Fstl1, Sparc, Tgfbr3, Col14a1, Lgals1, Antxr1, Axl, Fbn1

† These gene clusters refer to those highlighted on the x-axis in Fig. 3a; examples of prominently expressed genes in each cluster are listed.

Supplementary References

1. Fu, N.Y. *et al.* EGF-mediated induction of Mcl-1 at the switch to lactation is essential for alveolar cell survival. *Nat Cell Biol* **17**, 365-375 (2015).
2. Lim, E. *et al.* Transcriptome analyses of mouse and human mammary cell subpopulations reveal multiple conserved genes and pathways. *Breast Cancer Res* **12**, R21 (2010).
3. Sheridan, J.M. *et al.* A pooled shRNA screen for regulators of primary mammary stem and progenitor cells identifies roles for Asap1 and Prox1. *BMC Cancer* **15**, 221 (2015).
